# The Multilayered Landscape of Ferroptosis: Plasticity, Propagation, and Evolutionary Perspectives

**DOI:** 10.3390/antiox15010111

**Published:** 2026-01-15

**Authors:** Hong Chen, Hongfa Yan, Hong Bu, Feng Ye

**Affiliations:** 1 Department of Pathology and Institute of Clinical Pathology, West China Hospital, Sichuan University, Chengdu 610041, China; chenhong3323@126.com; 2 Department of Neurology and State Key Laboratory of Biotherapy, National Clinical Research Center for Geriatrics, West China Hospital, Sichuan University, Chengdu 610041, China; yyhyhff@foxmail.com

**Keywords:** ferroptosis, lipid peroxidation, antioxidant, propagation

## Abstract

Ferroptosis is a distinct form of regulated necrotic cell death driven by iron-dependent phospholipid peroxidation, characterized by flexible and context-dependent mechanisms rather than a single fixed linear pathway. This study elucidates the critical lipid peroxidation networks and antioxidant defense systems used in determining ferroptosis, specifically emphasizing how these mechanisms underpin the plasticity of this cell death mode and its correlation with therapeutic resistance. We examine the catastrophic propagation of ferroptosis, detailing the multi-layered amplification mechanisms—ranging from intracellular organelle crosstalk to intercellular trigger waves—that may facilitate massive tissue damage in degenerative diseases and ischemic injuries. Furthermore, the evolutionary conservation of ferroptosis-like phenomena across diverse species is summarized, underscoring its fundamental role in development and host–pathogen interactions. To conclude, we explore pivotal knowledge gaps that remain in our understanding of ferroptosis. By integrating these complex regulatory networks, this review provides a comprehensive framework for understanding ferroptosis as an adaptable, self-amplifying process, informing future efforts to modulate ferroptosis in disease contexts. Notably, this review focuses on the amplification, execution, and propagation phases of ferroptosis rather than on its initial triggering mechanisms, which remain an area of active investigation.

## 1. Introduction

Ferroptosis is a distinct form of regulated necrotic cell death characterized by iron-dependent phospholipid peroxidation, whose susceptibility is conserved and modulated by dysregulated iron metabolism and redox imbalance [1,2]. Accumulating evidence indicates that ferroptosis does not operate through a single obligatory molecular trigger, but instead emerges from the integration of multiple biochemical processes that vary across cellular and pathological contexts.

Two key observations exist in existing ferroptosis research: (1) although numerous metabolites and proteins can initiate or regulate ferroptosis, no single one is absolutely essential in all biological contexts; and (2) the same molecules can exhibit distinct pro- and anti-ferroptotic activities under different cell-type-specific signaling pathways and stress-dependent induction patterns. This operational paradigm requires defining ferroptosis as a death mode orchestrated by a combination of biochemical mechanisms in specific environments, rather than a strictly linear pathway. At the macromolecular level, ferroptosis fundamentally requires the catastrophic accumulation of lipid peroxides—a process that depends on three key elements: an oxidizable lipid substrate, molecular oxygen, and reactive iron to facilitate autocatalytic propagation through both enzymatic and non-enzymatic mechanisms [3]. These elements act as metabolic hubs, connecting interconnected networks encompassing lipid metabolism, iron homeostasis, antioxidant defense, and bioenergetics. The inherent complexity of these networks, coupled with tissue-specific regulatory pathways, endows ferroptosis with remarkable plasticity.

Beyond the single-cell level, ferroptosis has been increasingly associated with large-scale, progressive tissue injury and degenerative diseases [4,5,6], suggesting that ferroptosis undergoes a series of intracellular and extracellular amplification and diffusion events to support its feedback amplification. Understanding these feedback amplification mechanisms has the potential to prevent the progression of neurodegenerative diseases or induce ferroptosis in refractory cancers.

Importantly, lipid peroxidation itself is a fundamental biochemical process observed across a wide spectrum of species, ranging from plants and invertebrates to mammals, reflecting its deep evolutionary conservation in cellular physiology [7]. Because ferroptosis is defined by iron-dependent phospholipid peroxidation, this shared vulnerability of membrane lipids to oxidative damage raises the possibility that core features of ferroptosis are conserved across diverse biological systems.

This review will reveal the mechanistic plasticity and propagation characteristics of ferroptosis by dissecting the complex intracellular lipid peroxidation and antioxidant networks, and the spatiotemporally coordinated feedback and diffusion pathways, thereby advancing our understanding of ferroptosis. It will also summarize ferroptosis-like phenomena in various species, emphasizing their potential physiological relevance and evolutionary significance.

## 2. Mechanistic Plasticity: A Game Between Lipid Peroxidation and Antioxidant Networks

Cells typically possess multiple parallel signaling pathways to execute critical functions, ensuring robust survival. Many metabolites and proteins can initiate or regulate ferroptosis, yet not all are essential or sufficient within organisms. For example, acyl-CoA synthetase long-chain family member 4 (ACSL4) is widely considered as necessary for ferroptosis, and its mediated biosynthesis of polyunsaturated fatty acid (PUFA)-containing phosphatidylethanolamines (PEs-PUFA), which serve as preferential substrates for lipid peroxidation [8]. However, photodynamic therapy induces ferroptosis by directly peroxidizing PUFAs and generating exogenous reactive oxygen species (ROS) in an ACSL4-independent manner [9]. This phenomenon reveals profound redundancy and compensation mechanisms in cellular ferroptosis pathways. In addition, the same molecule may exhibit opposite regulatory effects on ferroptosis in different disease contexts and metabolic states. For instance, low-dose doxorubicin or short-term Nutlin treatment can induce p53 transactivation of calcium-independent phospholipase A_2_β (iPLA_2_β), thereby suppressing ferroptosis in melanoma and sarcoma cells [10]. In contrast, the earlier reports demonstrated that p53 can suppress solute carrier family 7 member 11 (*SLC7A11*) transcription, reduce glutathione levels and glutathione peroxidase 4 (GPX4) activity, and promote ferroptosis [11]. Recent genome-wide CRISPR–Cas9 screens identified 7-dehydrocholesterol (7-DHC) as an endogenous lipid factor that confers resistance to ferroptosis [12,13], Owing to a conjugated diene within its sterol core, 7-DHC efficiently scavenges lipid-derived radicals and terminates lipid peroxidation chain reactions. Functionally, 7-DHC acts as a sacrificial lipid antioxidant, preferentially undergoing oxidation to limit the propagation of oxidative damage to surrounding phospholipids, including ferroptosis-relevant PUFA-containing species. This mechanism highlights that lipid composition and intrinsic radical-trapping capacity modulate ferroptosis susceptibility. Together, these phenomena point to a core conclusion: ferroptosis is not a straightforward process controlled by a single linear pathway, but rather a highly dynamic, interconnected, and context-specific network of cell fate decisions.

### 2.1. Plasticity of Lipid Peroxidation

#### 2.1.1. Diverse Substrates for Lipid Peroxidation

The initiation of both enzymatic lipid peroxidation and autoxidation relies on sufficient “fuel” reserves within the plasma membrane. Experimental evidence has shown that deuterium substitution at bis-allylic positions effectively suppressed autoxidation, validating the critical role of PUFAs in this process [14]. Notably, oxidation of esterified PUFAs is a prerequisite for classical ferroptosis mechanisms, particularly PEs-PUFA [15,16]. The ACSL4-lysophosphatidylcholine acyltransferase 3 (LPCAT3) axis orchestrates PE-PUFA synthesis and represents the main pathway in GPX4 inhibition-induced ferroptosis [17]. However, its importance is diminishes in models such as cystine deprivation [18], p53 induction [19], and photodynamic therapy [20]. Beyond PEs-PUFA, other phospholipids including phosphatidylcholine, phosphatidylserine, and phosphatidylglycerol can also promote ferroptosis [21]. The differential oxidizability among various phospholipids arises because the headgroup influences membrane localization, chemical properties, and membrane conformation [22]. Moreover, Qiu et al. [21] demonstrated that diacyl-PUFA-containing phosphatidylcholine exhibit even greater potency in promoting ferroptosis compared to PEs-PUFA supplementation. In addition to the phospholipids mentioned, peroxisome-derived polyunsaturated ether phospholipids can also participate in lipid peroxidation and drive ferroptosis [23]. However, polyunsaturated ether phospholipids are not inherently more susceptible to peroxidation than polyunsaturated phospholipids, and it remains unclear whether they can functionally replace polyunsaturated phospholipids in promoting ferroptosis.

**Figure 1:**
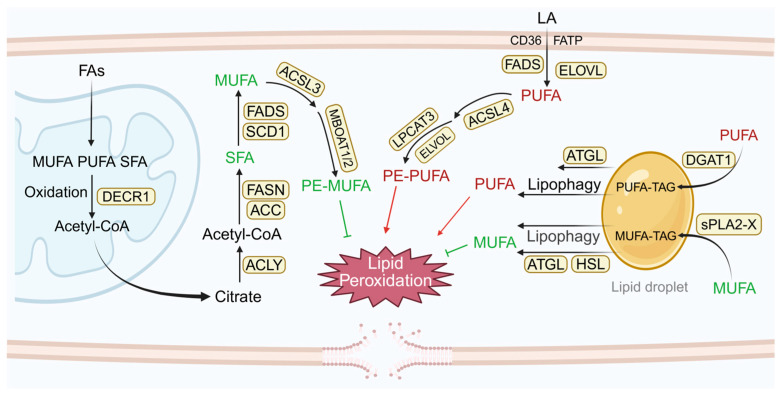
Ferroptosis-associated lipid peroxidation substrates.De novo fatty acid synthesis from acetyl-CoA via enzymes such as ACC and FASN yields SFA. SFA undergoes SCD1/FADS-mediated desaturation to form MUFA, which is acetylated by MBOAT1/2 and incorporated into membrane phospholipids via ACSL3. Exogenous PUFAs, such as linoleic acid (LA), are imported via transporters (CD36/FATP) and processed by ELOVL2/5 and FADS2/1, generating long-chain PUFAs. AA and AdA, critical substrates for ferroptosis, are acylated by LPCAT3 and integrated into membrane phospholipids/ether phospholipids via ACSL4. Exogenous PUFA increases DGAT1-dependent biogenesis of PUFA-TAG-rich lipid droplets. Subsequent ATGL/lipophagy-mediated diversion of PUFA to membrane phospholipids elevates lipid peroxidation. Concurrent increases in MUFA and PUFA lead to their accumulation in lipid droplets. Subsequent lipolysis via ATGL, HSL, and possibly lipophagy releases MUFA and PUFA, reducing the abundance of oxidizable PUFA in membranes and thereby limiting lipid peroxidation. ACC, acetyl-CoA carboxylase; FASN, fatty acid synthase; SCD1, stearoyl-CoA desaturase 1; FADS, fatty acid desaturase; MBOAT1/2, membrane-bound O-acyltransferase domain-containing 1 and 2; ACSL3/4, acyl-CoA synthetase long-chain family member 3/4; CD36, also known as fatty acid translocase (FAT), glycoprotein IIIb (GPIIIb), or glycoprotein IV; FATP, fatty acid transport protein; ELOVL, elongase; LPCAT3, lysophosphatidylcholine acyltransferase 3; DGAT1, diacylglycerol acyltransferase 1; TAG, triglycerides; ATGL, adipose triglyceride lipase; HSL, hormone sensitive lipase; sPLA_2_-X, Secreted phospholipase A_2_ group X. FAs: fatty acids; SFA, saturated fatty acid; MUFA, monounsaturated fatty acid; PUFA, polyunsaturated fatty acid; AA, arachidonic acid; AdA, adrenic acid. Created in BioRender. Jiao, L. (2026) https://BioRender.com/ikuxgia (accessed on 11 January 2026).

In contrast, the incorporation of oxidation-resistant monounsaturated fatty acids (MUFAs) into membrane phospholipids protects cells from ferroptosis. MUFAs are competitively incorporated into phospholipids through ACSL3-membrane-bound O-acyltransferase domain-containing 1 and 2 (MBOAT1/2), reducing the PUFA/MUFA ratio and thus inhibiting lipid peroxidation [24]. Lipid droplets (LDs) are storage organelles composed mainly of triglycerides and sterol esters. Nutritional and oxidative stressors modulate LD biogenesis to regulate membrane unsaturation through dynamic PUFA/MUFA partitioning between triglycerides and phospholipid pools, thereby influencing ferroptosis susceptibility [25]. The diacylglycerol acyltransferase 1 (DGAT1)-mediated PUFA channeling into LDs protects against oxidative stress-induced cell death under exogenous PUFA overload [26]. Conversely, LDs may also promote ferroptosis through either direct peroxidation or context-dependent release of PUFAs [27,28] (Figure 1).

Therefore, ferroptosis does not rely on a single lipid class or a fixed pathway. Instead, its execution mechanism adapts dynamically according to the substrate availability, abundance, and the cellular environment.

#### 2.1.2. Redundancy in Lipid Peroxidation Triggering Pathways

**Figure 2:**
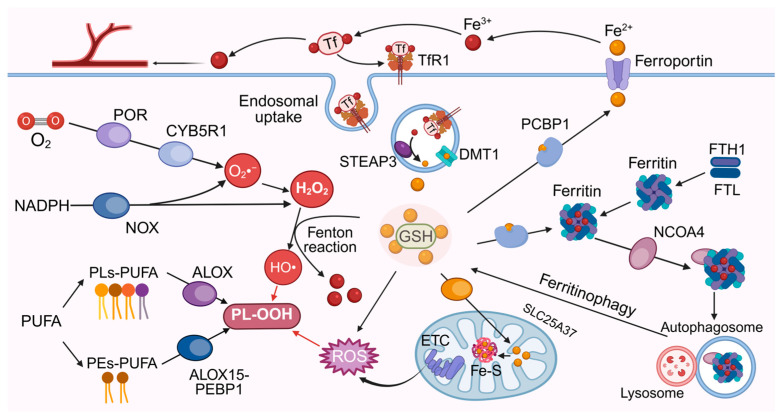
The ROS and iron metabolism in ferroptosis. Fe^3+^ bound to transferrin enters cells via TfR1-mediated endocytosis. Within endosomes, STEAP3 reduces Fe^3+^ to Fe^2+^, which is transported to the cytosol by DMT1. Labile iron can be sequestered by ferritin, whose synthesis and degradation regulate the labile iron pool (LIP). NCOA4 mediates ferritin degradation via ferritinophagy. Iron export is mediated by ferroportin in enterocytes and macrophages. Labile iron generates ROS via the Fenton reaction, promoting lipid oxidation. Mitochondrial electron transport chain (ETC) complexes and oxidases (e.g., ALOX, NOX, POR, CYB5R1) also generate ROS, catalyzing PUFA oxidation and lipid peroxidation. Tf, transferrin; TfR1, transferrin receptor 1; STEAP3, six-transmembrane epithelial antigen of prostate 3; DMT1, divalent metal transporter 1; NCOA4, nuclear receptor coactivator 4; PCBP1, poly(C)-binding protein 1; ALOX, arachidonate lipoxygenase; NOX, NADPH oxidase; POR, NADPH-cytochrome P450 oxidoreductase; CYB5R1, NADPH-cytochrome b5 reductase 1. Created in BioRender. Jiao, L. (2026) https://BioRender.com/orm50go (accessed on 11 January 2026).

The lipid peroxidation process consists of two phases: initiation (radical generation) and amplification (chain reaction). The initiation can proceed through multiple parallel routes: the non-enzymatic pathway generates ROS through the Fenton reaction mediated by ferrous ions (Fe^2+^) or mitochondrial electron leakage, which initiates lipid radicals and triggers an autocatalytic cascade reaction, constituting the core amplification mechanism of lethal accumulation of lipid peroxides [2,29]; In parallel, enzymatic pathways provide diverse initiation mechanisms, including oxidation of specific phospholipid substrates by arachidonate lipoxygenase (ALOX) family [30], NADPH-dependent hydrogen peroxide production by oxidoreductase NADPH-cytochrome P450 reductase (POR)/NADH-cytochrome b5 reductase (CYB5R1) [31], and tissue-specific ROS generation by NAD(P)H oxidase (NOX) family [32] (Figure 2). Enzymatic lipid peroxidation may predominantly function during the initiation phase, while subsequent amplification largely relies on non-enzymatic autoxidation. For example, in type II ferroptosis inducer (RSL3)-induced ferroptosis, intracellular peroxidized lipids surge within 1 h of treatment, culminating in cell death within 4 h [18]. If enzymatic processes dominated this rapid progression, such dramatic lipid peroxidation kinetics would require concurrent upregulation of peroxidation-related enzymes, but this phenomenon not supported by empirical evidence.

Different triggers may activate distinct dominant pathways to induce ferroptosis. In the R6/1 Huntington’s disease model, loss of ALOX5 expression eliminates HTTQ94-associated ferroptosis under ROS or glutamate stress and ameliorates pathological phenotypes [33]. Meanwhile, NOX2 plays a critical role in stress-induced ferroptosis in retinal ganglion cells [34]. Similarly, different enzymatic pathways predominate in different cell types. Inhibition of ALOX5 alleviates dopaminergic neuronal ferroptosis and motor deficits in a Parkinson’s disease mouse model [35], and its protective role extends to mouse models of stroke [36] and epilepsy [37]. On the other hand, the NOX family may play a more prominent role in tumor contexts. For instance, NOX2 and NOX4 enhance ferroptosis in epithelial ovarian cancer and renal malignancies, respectively [38,39]. Notably, significant functional redundancy exists among these enzymatic pathways, as evidenced by the fact that the loss of ALOX can be compensated for by POR or non-enzymatic mechanisms [2,40,41].

Additionally, iron bioavailability is a key determinant of ferroptosis. As an essential enzymatic cofactor and catalyst in Fenton reactions, iron dynamically integrates the efficiency of various pathways through networks involving transferrin receptor 1 (TfR1)-mediated endocytosis, ferritin storage, and nuclear receptor coactivator 4 (NCOA4)-mediated ferritinophagy [42]. Consequently, the iron regulatory network, encompassing uptake, storage, export, and intracellular utilization, critically governs ferroptosis dynamics [42] (Figure 2). This multi-tiered regulatory system underpins the remarkable plasticity of ferroptosis. It enables the sustained maintenance of lipid peroxidation through the release of stored iron from ferritin, even in the face of impaired iron uptake, thereby guaranteeing the adaptive propagation of death signals across diverse stress conditions.

### 2.2. Plasticity of the Antioxidant Defense System

#### 2.2.1. Core Defense Systems and Compensatory Mechanisms

**Figure 3:**
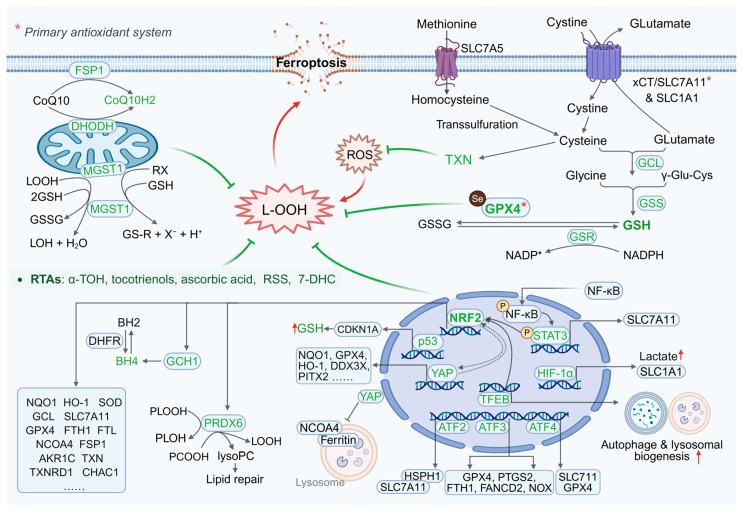
The antioxidant defense system. Key intracellular protective pathways/enzymes include: xCT-GSH-GPX4, FSP1-CoQ10, PRDX6, GCH1-BH4-DHFR, DHODH-CoQH2, and MGST1. Transcription factors (NRF2, ATFs, NF-κB, p53, YAP, STAT3, TFEB, HIF-1α) cooperatively regulate defense against lipid peroxidation. In addition, this protection can also be achieved through endogenous radical-trapping antioxidants (RTAs) and modulators of key lipid metabolism/antioxidant enzymes. Red upward arrows indicate an increase in the preceding molecules or compounds. The ellipses indicate that other targets exist beyond those listed in the diagram. xCT/SLC7A11, solute carrier family 7 member 11; SLC1A1, solute carrier family 1 member 1; SLC7A5, solute carrier family 7 member 5; GCL, glutamate cysteine ligase; TXN, thioredoxin; GSS, glutathione synthetase; GSH, glutathione; GPX4, glutathione peroxidase 4; GSR, glutathione reductase; GSSG, oxidized glutathione; L-OOH, lipid hydroperoxide; α-TOH, α-tocopherol; RSS, reactive sulfur species; 7-DHC, 7-dehydrocholesterol; FSP1, Ferroptosis suppressor protein 1; DHODH, dihydroorotate dehydrogenase; MGST1, microsomal glutathione S-transferase 1; NRF2, nuclear factor erythroid 2-related factor 2; STAT3, signal transducer and activator of transcription 3; YAP, Yes-associated protein; HIF-1α, hypoxia inducible factor-1 alpha; TFEB, transcription factor EB; ATF, activating transcription factor; PRDX6, peroxiredoxin 6; GCH1, GTP cyclohydrolase 1; BH4, tetrahydrobiopterin; DHFR, dihydrofolate reductase. Created in BioRender. Jiao, L. (2025). https://BioRender.com/17xtzwo (accessed on 30 December 2025).

To cope with oxidative damage, cells possess a robust and multi-layered antioxidant defense system (Figure 3). Within this system, GPX4 serves as the central defense against ferroptosis by uniquely catalyzing the reduction in phospholipid hydroperoxides [43]. The loss or inhibition of GPX4 can be partially compensated for by parallel antioxidant mechanisms [44]. Membrane-localized ferroptosis suppressor protein 1 (FSP1) utilizes NAD(P)H to reduce ubiquinone to ubiquinol (CoQH_2_), acting as a lipophilic radical-trapping antioxidant (RTA) to directly quench lipid radicals [45]; peroxiredoxin 6 (PRDX6) scavenges short-chain hydroperoxides through its peroxidase activity and hydrolyzes phospholipid hydroperoxides to generate lysophospholipids through phospholipase A_2_ (PLA_2_) activity, thereby synergistically limiting membrane damage propagation [46]; metabolic networks such as the GTP cyclohydroxylase 1 (GCH1)-tetrahydrobiopterin (BH4) axis neutralize lipid peroxyl radicals via BH4 [47], while enzymes like aldo-keto reductase family 1 member C1 (AKR1C1) detoxify cytotoxic aldehyde byproducts derived from lipid peroxidation [48]. Although these systems do not directly reduce phospholipid hydroperoxide, they dynamically establish compensatory defenses upon GPX4 dysfunction by limiting radical accumulation, removing oxidized lipid species, and detoxifying secondary metabolites. This “core is irreplaceable—bypass pathways are compensatory” architecture confers significant environmental adaptability to ferroptosis regulation, allowing cells to selectively mobilize dominant antioxidant routes to maintain redox homeostasis.

#### 2.2.2. Diversity of Antioxidant Molecules

Some endogenous molecules can provide rapid protection by directly quenching lipid radicals or interrupting peroxidation chain reactions [49]. For instance, vitamin E (e.g., α-tocopherol) [50] and reduced coenzyme Q10 (CoQ10H_2_) [51,52] directly quench lipid peroxyl radicals through hydrogen atom transfer, anchoring within biological membranes to provide immediate protection. Beyond vitamin E, other vitamins have also been reported to have antioxidant effects [53]. Vitamin K forms (specifically menaquinone-4) exhibit potent radical-neutralizing capabilities [54].Vitamins A and D primarily modulate antioxidant gene expression via binding to specific nuclear receptors, indirectly conferring protection [55,56,57,58]. In addition, ascorbic acid (also known as vitamin C) can prevent ferroptosis through the nuclear factor erythroid 2-related factor 2 (NRF2) and SLC7A11-GPX4 pathways [59,60]. H_2_S-derived reactive sulfur species (RSS) can diffuse intracellularly to scavenge a variety of radicals by virtue of their strong reducing capacity [61]. Recent reports have also found that the presence of 7-DHC with a B-ring diene structure is a highly efficient RTA on the mitochondrial membrane [12,13]. Additionally, even molecules present at low concentrations, such as BH4, contribute by efficiently quenching superoxide and lipid radicals [62].

These molecules enable spatial complementarity within the antioxidant defense network. Specifically, vitamin E and CoQ10H_2_ synergistically protect the plasma membrane [63]; vitamin A-related retinoids contribute both direct antioxidant buffering at membranes and longer-term transcriptional reinforcement of ferroptosis defense programs [58]; 7-DHC safeguards mitochondrial and other cellular membranes [12], and RSS scavenges radicals within the cytosol [64]. When subjected to specific stressors, cells mobilize the most appropriate molecules to mount a primary defense and elicit synergistic protection. Vitamin E/CoQ10H_2_ help maintain basal redox homeostasis, while RSS offers broad scavenging capacity during ROS bursts. Furthermore, retinoids integrate redox control with lipid remodeling and the induction of ferroptosis-resistance gene, while 7-DHC is activated in response to mitochondrial stress. Therefore, cells flexibly call on optimized defense strategy according to the intensity of oxidative stress, subcellular damage sites, and metabolic status, highlighting the dynamic adaptability of the ferroptosis defense network.

#### 2.2.3. Transcriptional Remodeling of Antioxidant Defenses

Transcriptional regulators confer plasticity in ferroptosis resistance by dynamically modulating antioxidant defense pathways. As a master regulator, NRF2 maintains redox and iron metabolism homeostasis by upregulating antioxidant enzymes (e.g., GPX4, SLC7A11, FSP1) [65,66] and lipid peroxidation inhibitors (e.g., AKR family members, PPARG) [67,68] (Figure 3). Its targeted inhibitors plumbagin or ML385 can enhance ferroptosis sensitivity in cancer therapy [69,70,71,72], while activators salidroside or sulforaphane play a protective role in neurodegenerative and metabolic diseases [73,74]. The activating transcription factor (ATF) family members (ATF1-4) regulate cell ferroptosis in a context-dependent manner by recognizing and binding to cAMP response elements (CREs) and influencing the transcription of multiple target genes [75]. Of which, ATF2 enhances antioxidant defense by stabilizing SLC7A11 [76], ATF3 inhibit SLC7A11/GPX4 to promote ferroptosis [77,78], and can also be regulated by NRF2 to enhance glutathione protection [79]. ATF4 inhibits lipid peroxidation by inducing the heat shock protein family A member 5 (HSPA5)-GPX4 axis [80], upregulating SLC7A11 [81], or activating the nuclear protein 1 (NUPR1)-lipocalin 2 (LCN2) pathway [82], and cooperates with NRF2 and YAP/TAZ to maintain redox homeostasis [83,84]. As a member of the nuclear factor of κ-light chain of enhancer-activated B cells (NF-κB) family, NF-κB p65 suppresses ferroptosis by directly regulating genes related to iron accumulation and/or lipid peroxidation [85]. Specifically, it transactivates SLC7A11 [86], induces LCN2 [87] and upregulates antioxidant proteins thioredoxin, heme oxygenase 1 (HO-1), and ferritin heavy chain 1 (FTH1) [88], and its phosphorylation level is associated with chemotherapy resistance [87]. Similarly to ATF3, p53 also plays a dual role in regulating ferroptosis. It promotes ferroptosis by inhibiting stearoyl-CoA desaturase-1 (SCD1) [89] and MBOAT1 [90] or upregulating ACSL4 [91], yet also enhances antioxidant defense via phospholipid transfer protein (PLTP)-mediated PUFA sequestration [92], induction of cyclin-dependent kinase inhibitor 1A (CDKN1A) expression [93], and suppression of the mevalonate pathway [94,95]. Furthermore, other factors can also modulate ferroptosis sensitivity to varying extents by remodeling the antioxidant network. YAP functions by cooperating with NRF2 [96], upregulating GPX4 [97], and blocking ferritinophagy [98]. Signal transducer and activator of transcription 3 (STAT3), through inducing SLC7A11 and promoting the incorporation of PUFAs into triglycerides, also plays a role [99,100]. The transcription factor EB (TFEB) contributes by activating the autophagy-NRF2 axis and maintaining iron homeostasis [101,102]. Additionally, hypoxia inducible factor-1 alpha (HIF-1α) enhances antioxidant genes via the NF-κB/Wnt pathway, further influencing ferroptosis regulation [103].

In summary, transcriptional factors underpin the plasticity of ferroptosis regulation via multi-layered control of key antioxidant and lipid peroxidation nodes. Their functions are highly dependent on disease context, microenvironment, and stress conditions. Precisely targeting specific transcriptional factors may offer novel therapeutic strategies for cancer and degenerative diseases.

## 3. Propagation: The Self-Catalyzing Storm of Lipid Peroxidation

Previous study has indicated a connection between ferroptosis and degenerative diseases characterized by extensive contiguous tissue damage [4]. As early as 2014, research by Linkermann et al. [104] demonstrated that, unlike control mice which all died within 48 to 72 h following 40 min of ischemia prior to reperfusion, Fer-1-treated mice did not develop functional acute renal failure or structural organ damage after ischemia–reperfusion injury (IRI). This suggests that ferroptosis mediates rapid and large-scale tubular cell death in renal IRI. Widespread contiguous spread of excessive cell death has also been observed across tissues in other models of IRI and degenerative disorders [105,106], implying that ferroptosis may can be amplified by a series of feedback/amplification loops that enable the rapid and sustained propagation of cell death events.

### 3.1. Intracellular: Multi-Layered Propagation Mechanisms

#### 3.1.1. Radical Chain Reaction

The core propagation mechanism of ferroptosis originates from the radical chain reaction in non-enzymatic lipid peroxidation. The process is initiated when Fe^2+^ converts hydrogen peroxide into hydroxyl radicals via the Fenton reaction or when hydroxyl radicals are generated from superoxide dismutation derived from mitochondrial electron leakage [2,29,107]. In addition to these redox-driven routes, ferroptosis initiation can also occur through oxidase-mediated mechanisms, in which multiple oxidoreductases actively generate reactive oxygen species to initiate lipid peroxidation prior to extensive radical chain propagation [108]. Hydroxyl radicals selectively abstract the diallyl hydrogen atoms of PUFAs, forming lipid radicals. Lipid radicals rapidly react with molecular oxygen to generate highly reactive lipid peroxyl radicals, which propagate the chain by abstracting hydrogen from adjacent PUFAs, generating lipid hydroperoxides and regenerating lipid radicals. Lipid hydroperoxides further react with Fe^2+^ to generate lipid alkoxyl radicals, which rearrange to form epoxy peroxide radicals and regenerate peroxyl radicals, triggering a secondary peroxidation cascade [109]. The inherent instability of lipid hydroperoxides, peroxyl radicals, and lipid alkoxyl radicals lead to continuous production of oxidative products. Through a cyclic regeneration process, a self-sustaining feedback loop is established, resulting in exponential accumulation of lipid peroxides [30]. The intrinsic property of the radical reaction provides the fundamental driving force for the propagation nature of ferroptosis.

#### 3.1.2. Autophagy-Driven Propagation of Ferroptosis

**Figure 4:**
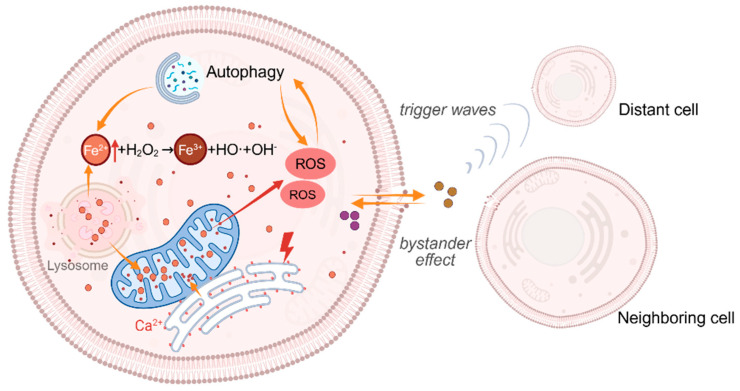
Feed-Forward Propagation of ferroptosis. During ferroptosis, increased ROS induce autophagy, which further promotes the accumulation of iron and ROS (e.g., through ferritinophagy or mitophagy). Meanwhile, inter-organellar ion transfer disrupts organellar homeostasis and function, ultimately leading to bursts of mitochondrial and cytosolic ROS and enhanced lipid peroxidation. Additionally, ions, signaling molecules, and peroxidation products (such as truncated lipids) generated in this process can propagate in a wave-like manner through the cell population, thereby inducing death in additional cells. The red upward arrows indicate an increase. Created in BioRender. Jiao, L. (2025). https://BioRender.com/b7lemgc (accessed on 30 December 2025).

Growing evidence indicates that autophagy plays an important regulatory role in ferroptosis. This is supported by the observation of increased autophagy in erastin-induced mouse embryonic fibroblasts and HT1080 cells [110]. The fact that N-acetylcysteine treatment can block the induction of autophagy during ferroptosis, suggesting that autophagy may be triggered by high levels of intracellular ROS [110]. Additional evidence also shows that ROS accumulated during the early stages of ferroptosis activate pathways such as ataxia-telangiectasia mutated (ATM) kinase (a DNA damage response transducer) [111], mitogen-activated protein kinase (MAPK) [112], and the mechanistic target of rapamycin (mTOR) [113], which promote the expression of autophagy-related genes and initiate autophagic flux. Crucially, activated autophagy can further exacerbate lipid peroxidation through multiple effector pathways. For example, NCOA4-mediated ferritinophagy releases stored iron [114], while lipophagy degrades LDs to supply PUFA substrates [115,116]. Consistent with this, pharmacological inhibition of autophagy (BafA1/chloroquine) can reduce erastin/FIN56-induced ferroptosis and attenuates ROS increases triggered by ferroptosis inducers [117,118]. Thus, autophagy acts as a key bridging mechanism in the propagation of ferroptosis, forming a self-catalytic cycle of ROS increase and autophagy activation (Figure 4).

#### 3.1.3. Inter-Organellar Crosstalk and Cascading Amplification

During ferroptosis, a series of physiological and biochemical reactions occur in the cell. These take place both in the cytosol and within organelles of the endomembrane system, including mitochondria, endoplasmic reticulum (ER), lysosomes, Golgi apparatus and peroxisomes. Alterations in the structure or function of these organelles, along with inter-organellar crosstalk, can synergistically promote the amplification of damage or death signals.

ER-mitochondria crosstalk constitutes an early driving hub in ferroptosis. Study has shown that ferroptosis inducers (e.g., RSL3) can trigger the dynamic remodeling of ER-mitochondrial contact sites (EMCSs) within minutes, including a significant expansion of the contact area and a shortening of the membrane distance [119]. This remodeling allows oxidized phospholipids (especially oxidized phosphatidylethanolamine and phosphatidylcholine) generated at this interface to be rapidly transferred to the mitochondrial membrane, leading to mitochondrial ROS generation and dysfunction [119]. Meanwhile, ROS derived from the mitochondrial respiratory chain can disrupt Ca^2+^ homeostasis in the ER, thereby hindering protein synthesis and release [120]. A large number of unfolded and misfolded proteins accumulate and cause ER stress. The ER subsequently releases Ca^2+^ into the mitochondria and cytoplasm, causing mitochondrial Ca^2+^ overload and further ROS production, forming an enhanced loop (Figure 4). In this process, proteins such as mitofusin 2 (MFN2) and its splice variant ERMIT2 regulate EMCS stability [121], while optic atrophy 1 (OPA1) affects the efficiency of ROS generation by maintaining the mitochondrial cristae structure and regulating the spatial structure of EMRCs [122], collectively determining the magnitude of cascade amplification.

Unlike the ER-mitochondria crosstalk, lysosome-mitochondria crosstalk provides a critical axis for iron metabolism amplification in ferroptosis (Figure 4). Lysosomes accumulate iron ions through the autophagic degradation of iron-rich proteins and cellular debris [123]. Increased lysosomal membrane permeability (e.g., after 4 h of RSL3 treatment) leads to rapid leakage of Fe^2+^ into the cytosol, directly catalyzing the Fenton reaction and driving lipid peroxidation [124]. Additionally, a “kiss and run” mechanism supports direct Fe^2+^ transfer from lysosomes to mitochondria [125]. This process involves membrane contact sites [126] and Fe^2+^ transport mediated by the divalent metal transporter 1 (DMT1)/the voltage-dependent anion channel 1 (VDAC1) located on the lysosomal and mitochondrial outer membranes [127]. Subsequently, mitoferrin2 on the mitochondrial inner membrane efficiently transports Fe^2+^ into the mitochondrial matrix, providing substrate for ROS burst and promoting the generation of toxic lipid radicals [128]. Furthermore, impaired mitochondrial function adversely affects lysosomal activity and acidification [129]. More critically, mitochondrial ROS can further elevate cellular iron levels via the autophagy-lysosome pathway, thereby activating ferroptosis [130]. Thus, iron trafficking between lysosomes and mitochondria drives a self-sustaining propagation of disrupted iron homeostasis and oxidative damage.

### 3.2. Intercellular: Synergistic Amplification via Contact-Dependent and Non-Contact Trigger Waves

Accumulating evidence from both in vitro and in vivo studies indicates that ferroptosis exhibits non-random, wave-like transmission characteristics in tissues [131,132]. Its mechanism includes two synergistic amplification pathways: contact-dependent lipid peroxidation transfer and non-contact trigger waves. The former occurs at cell–cell contact interfaces and relies on iron ions and oxidizable membrane lipids to extend the chain reaction of lipid peroxidation autocatalytically to adjacent plasma membranes, without the participation of intracellular components [133]. The latter originates from the damage of lipid peroxidation to the integrity of the cell plasma membrane, leading to cell swelling and the release of intermediate signaling molecules [132]. These molecules form self-sustaining trigger waves that can propagate long distances (>118 μm) without attenuation, inducing lipid peroxidation bursts and ferroptosis in neighboring cells [134].

It is worth noting that these two propagation modes exhibit selective dependence on the ferroptosis induction mechanism. Contact propagation has been demonstrated in the GPX4 inhibition model, but its universal chemical basis—lipid peroxidation being the core event in ferroptosis—suggests its potential broad applicability [18]. In contrast, trigger wave propagation has only been reported to occur under the condition of cysteine deficiency. Compared with the rapid lipid peroxidation initiation (within 1.5 h) in the GPX4 inhibition model, the slower progression and extensive ROS accumulation in erastin-induced ferroptosis provide the necessary time window and material basis for the production and release of intermediate mediators [135]. Although the exact molecular mediator remains elusive, key experimental evidence indicates that ROS is a diffusible signal: (1) conditioned medium containing factors secreted by ferroptotic cells can induce ferroptosis in recipient cells, indicating the presence of diffusible molecules; (2) cell death is greatly inhibited after pretreatment of this medium with ROS scavengers [134]. Consequently, the ferroptosis signal not only amplifies within cells but also propagates intercellularly via direct cell contact or diffusive molecules such as ROS, leading to widespread cell damage and population-level cell death.

## 4. Evolutionary Conservation in Development and Disease

**Figure 5:**
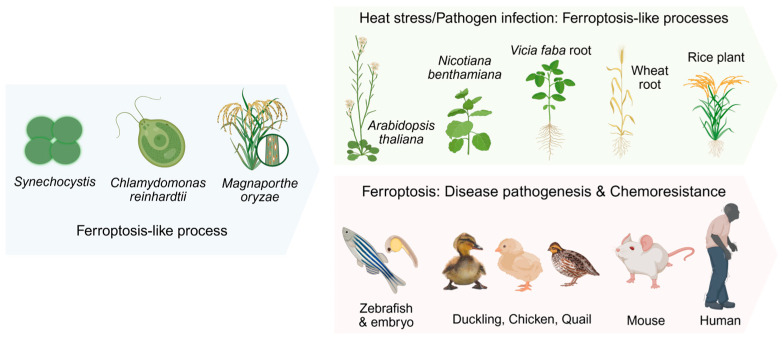
Evolutionary conservation of ferroptosis. The antioxidant defenses and ferroptosis(-like) biological characteristics are evolutionarily conserved, observed from prokaryotes (*Synechocystis* sp.) and unicellular eukaryotes (*Chlamydomonas reinhardtii*) to fungi (*Magnaporthe oryzae*), plants (*Arabidopsis thaliana*, tobacco, faba bean, wheat, rice), fish (zebrafish), birds (duckling, chicken, quail), and mammals (mouse, human). Created in BioRender. Jiao, L. (2025). https://BioRender.com/o8klivl (accessed on 30 December 2025).

To counteract oxidative damage, cells have evolved adaptive mechanisms to regulate intracellular lipid peroxidation. Lipid peroxidation is a radical-mediated cascade reaction in which molecular oxygen incorporates into lipids, generating lipid hydroperoxides via the peroxyl radicals’ intermediates. This process is ubiquitous in biological systems. Ferroptosis a form of iron-dependent regulated cell death driven by lipid peroxidation, is therefore hypothesized to be evolutionarily conserved across species (Figure 5).

Extensive studies in mouse models have firmly linked ferroptosis to the pathogenesis of multiple diseases, most prominently neurodegenerative disorders driven by pathogenic protein mutations. In models expressing SOD1^G93A^, TDP-43, or C9orf72, reduced GPX4 activity is tightly associated with ferroptotic lipid peroxidation, whereas genetic or pharmacological enhancement of GPX4 function significantly delays disease progression [136]. In Huntington’s disease mouse models driven by mutant huntingtin, pharmacological inhibition of ferroptosis with Liproxstatin-1 markedly reduces medium spiny neuron loss [137], while genetic deletion of *ALOX5* suppresses ferroptosis, rescues disease phenotypes, and prolongs survival [33]. Synucleinopathies, which are neurodegenerative disorders characterized by the pathological aggregation of α-synuclein and include Lewy body disease, Parkinson’s disease, and multiple system atrophy, provide additional evidence linking ferroptosis to protein-misfolding neurodegeneration. Multiple mouse studies have demonstrated that aberrant α-synuclein accumulation can directly induce neuronal ferroptosis [138,139]. Consistently, in human iPSC-derived neurons, overexpression of SNCA, which encodes α-synuclein, significantly reduces cellular viability. Notably, ferroptosis inhibitors—including deferoxamine (an iron chelator), deuterated polyunsaturated fatty acids, and Ferrostatin-1—substantially attenuate α-synuclein oligomer-induced neuronal death and restore cellular function [140]. Further support for a causal role of ferroptosis in neurodegeneration comes from neuroferritinopathy, a genetic disorder caused by ferritin mutations. These mutations lead to profound iron dysregulation and robust activation of ferroptosis, which can be effectively mitigated by ferroptosis inhibitors [141]. Importantly, human genetic evidence underscores the central role of GPX4 in neuronal ferroptosis suppression. In Sedaghatian-type spondylometaphyseal dysplasia, the disease-causing *GPX4^R152H^* mutation disrupts membrane targeting of GPX4. Despite retaining enzymatic activity, the mutant protein fails to localize properly to cellular membranes, rendering it incapable of suppressing lipid peroxidation. This defect results in uncontrolled neuronal ferroptosis and severe early-onset neurodegeneration [142], highlighting that GPX4-mediated ferroptosis protection critically depends on both enzymatic function and subcellular localization.

Beyond degenerative disorders, ferroptosis has emerged as a key determinant of therapeutic resistance in cancer, where reinforced antioxidant defenses and lipid metabolic remodeling enable tumor cells to escape ferroptosis under treatment pressure [128,129]. In parallel, ferroptosis plays a prominent role in inflammation and immune regulation, influencing immune cell survival, and inflammatory signaling [128,130]. Ferroptosis is also repeatedly implicated in murine ischemia–reperfusion injury, in which its inhibition confers substantial tissue protection [143]. Together, these disease models-many of which recapitulate core features of human pathology-support a conserved role for ferroptosis across neurodegeneration, cancer, inflammation, and acute tissue injury.

Recent studies have extended the relevance of ferroptosis beyond mammals to evolutionarily distant organisms. In Caenorhabditis elegans, aging is accompanied by pronounced ferroptotic features, and pharmacological inhibition of ferroptosis significantly extends lifespan [144]. Similarly, ferroptosis can be readily detected in *C. elegans* under specific stress conditions, including iron overload and dietary enrichment with PUFAs [145,146]. These observations indicate that the coupling between iron metabolism, PUFA oxidation, and ferroptotic cell death represents a deeply conserved biological vulnerability. The yolk of oviparous species is rich in PUFAs, which are essential for neural development but are highly susceptible to oxidative damage. To mitigate peroxidation-induced radical generation and lipid hydroperoxides formation, the oocyte system contains a complex array of antioxidants, including α-tocopherol [50,147], selenium [148], and antioxidant peptides, referring to small endogenous or dietary peptides capable of scavenging lipid radicals or supporting redox buffering [149]. The bioavailability of these nutrients is crucial during the development of oviparous organisms. For instance, selenium deficiency induces lipid peroxidation-related muscular dystrophy in chickens [150], while vitamin E deficiency in zebrafish embryos leads to dysregulation of thiols, amino acids, and related molecules [151], exacerbating lipid peroxidation and even leading to embryonic lethality [152]. Moreover, pharmacological inhibition of thiol-containing molecules can induce histological abnormalities and elevated malondialdehyde levels in zebrafish gonadal tissues [153]. Notably, studies have shown that broiler chickens under heat stress exhibit significant overproduction of ROS [154], which induces visceral oxidative damage [155] and activation of ferroptosis [156,157]. Emerging evidence also links ferroptosis to pathological manifestations of other species: novel duck reovirus (NDRV)-induced splenic lesions in ducklings and di(2-ethylhexyl) phthalate (DEHP)-mediated nephrotoxicity in quail (*Coturnix japonica*) [158,159]. These findings suggest that antioxidant defenses and ferroptosis pathways are involved in physiological and pathological processes in oviparous species.

Ferroptosis-like cell death processes have been widely reported in plants, such as *Arabidopsis thaliana* [160], rice plants [161,162], tobacco plants (*N. benthamiana*) [163], wheat roots (*Triticum aestivum* L.) [164], and faba bean roots (*Vicia faba*) [165] under heat stress and pathogen infection. Plant ferroptosis shares conserved molecular hallmarks with animal ferroptosis, including iron-dependent ROS accumulation, glutathione depletion, and lipid peroxidation, as well as morphological features such as cytoplasmic condensation, intact nuclei, and mitochondrial shrinkage [160]. In plants, ferroptosis-like cell death is generally considered maladaptive under abiotic stress conditions but may play a beneficial role in limiting pathogen spread during host–pathogen interactions, highlighting its context-dependent functional significance. While several reviews have outlined the landscape of ferroptosis in plants, a critical knowledge gap remains: the mechanisms governing ferroptosis-related signaling within and/or across species—particularly in host–pathogen interactions such as rice and *M. oryzae*—require systematic investigation to elucidate their regulatory networks and propagation dynamics [166,167,168].

Beyond plants, ferroptosis-like processes have also been observed in prokaryote *Synechocystis* sp. PCC 6803 [169] (which contains PUFA-rich thylakoid membranes despite prokaryotes generally lacking PUFAs in their membranes), unicellular algae *Chlamydomonas reinhardtii* [170], and fungal pathogens *Magnaporthe oryzae* [171]. Reviews by Conrad et al. [172] and Berndt et al. [173] described the roles and regulatory mechanisms of lipid peroxidation, ferroptosis, and antioxidant networks in yeast, bacteria, archaea, *trypanosoma brucei*, and *drosophila melanogaster.*

Although broader phylogenetic validation is warranted, current evidence underscores the evolutionary conservation of ferroptosis, which is involved in critical biological processes such as developmental regulation, stress adaptation, disease pathogenesis, and drug responses. These conserved pathways highlight ferroptosis as a universal cell death modality with profound implications for both fundamental biology and translational research.

Evidence suggests that while the ferroptosis program is explicitly defined in mammals, the core machinery of lipid peroxidation and associated antioxidant pathways display significant evolutionary conservation across plants, oviparous animals, and even microorganisms, indicating its ancient and fundamental role in biology.

## 5. Discussion

Ferroptosis, a regulated form of cell death, has emerged as an active field of research characterized by several distinctive features. Mechanistically, the dynamic interplay between lipid peroxidation and the antioxidant defense network confers plasticity to ferroptosis. lipid peroxidation can be initiated through different pathways and is influenced by substrate availability, whereas the antioxidant system adapts dynamically through a core-compensatory dual hub, expansion of the antioxidant molecule pool, and transcriptional reprogramming. The plasticity enables cells to maintain redox homeostasis under stress or nutrient imbalance, but also constitutes a foundation for drug resistance. Combination therapeutic strategies that simultaneously target multiple key nodes hold promise for overcoming current treatment limitations. Intracellular and extracellular Lipid peroxidation and ferroptosis-related signals/molecules form a spatially coordinated network, amplifying cell death effects exponentially across cell populations. This propagation system explains the widespread propagation of tissue damage and rapid pathological progression, highlighting its promise for novel therapeutic interventions in diseases like IRIs and neurodegenerative disorders. From an evolutionary perspective, ferroptosis(-like) and antioxidant processes are widely conserved across mammals, oviparous species, plants, and microorganisms, contributing to developmental homeostasis and host–pathogen interactions. These insights may inspire novel strategies for managing infectious diseases or modulating anti-tumor immunity.

Growing evidence suggests that in specific histological contexts or pathological states characterized by metabolic hyperactivity, lipid enrichment, or iron overload, ferroptosis may play a more prominent or earlier role compared to other regulated cell death (RCD) modalities, such as apoptosis, necroptosis, and pyroptosis [174]. In acute organ failure and IRI, ferroptosis often emerges as a prominent contributor [175,176,177]. Particularly in the kidneys and heart, the reperfusion-induced oxidative burst directly triggers membrane lipid peroxidation, leading to cell death before the activation of apoptotic pathways [178,179]. Studies in models of acute kidney injury, myocardial infarction, and stroke have demonstrated that inhibiting ferroptosis significantly attenuates tissue damage and improves functional recovery [180,181,182]. This is particularly salient in organ transplantation, where ferroptosis serves as a major lethal event post-reperfusion, positioning it as a promising therapeutic target [176]. Similarly, the brain is inherently susceptible to ferroptosis due to its high concentration of PUFAs and substantial accumulation of iron [183]. In neurodegenerative disorders like Parkinson’s and Alzheimer’s, aberrant protein aggregation, dysregulated iron metabolism, and chronic oxidative stress provide a sustained impetus for ferroptotic signaling [184]. Furthermore, mesenchymal-state or mutant (e.g., *KRAS*, *TP53*) cancer cells exhibit hypersensitivity to ferroptosis [11,185,186]. Metabolic reprogramming during epithelial–mesenchymal transition allows drug-resistant cells to evade apoptosis while simultaneously creating a ferroptosis-sensitive vulnerability, offering a strategy for treating recalcitrant malignancies like glioma [187,188,189].

Nevertheless, caution is warranted regarding the conclusion that ferroptosis is “dominant.” Ferroptosis is not an independent process but is deeply embedded within a complex regulatory network of RCD. Central to this network are ROS, which act as both the primary executioners of ferroptosis and the essential signaling conduits between diverse RCD pathways [190,191]. The accumulation of lipid-derived ROS triggers ferroptosis, whereas ROS compartmentalization and flux levels dictate the initiation of apoptosis, autophagy, necroptosis, or pyroptosis [192,193,194,195]. Concurrently, several key molecules function as “hubs” connecting different death pathways. For example, *TP53*, a classical pro-apoptotic gene, promotes apoptosis by activating p53 upregulated modulator of apoptosis (PUMA), Phorbol-12-myristate-13-acetate-induced protein 1 (PMAIP1), and other effectors [196,197]. It also acts as a multifaceted fate determinant by suppressing SLC7A11 to sensitize cells to ferroptosis, upregulating necrosis-related factor (NRF) to facilitate necroptosis, and activating expressions of pyroptosis regulators such as the NACHT, LRR and PYD domains-containing protein 3 (NLRP3), caspase 1, and gasdermin E (GSDME) [198]. Therefore, the interactions between ferroptosis and other cell death modalities are diverse and context-dependent. Specifically, ferroptosis and apoptosis exhibit a complex interplay of competition and synergism. On one hand, fatty acid oxidation activates STAT3 through CoA acetylation and upregulates the expression of ACSL4, a key ferroptosis-relate enzyme, enabling tumor cells to evade chemotherapy-induced apoptosis [199]. On the other hand, ferroptosis-induced mitochondrial dysfunction and ROS accumulation are known to trigger mitochondrial outer membrane permeabilization (MOMP), a definitive hallmark of the intrinsic apoptotic pathway [200]. Consequently, clinical strategies now increasingly aim to co-induce these two pathways to overcome therapeutic resistance [201]. Furthermore, ferroptosis and necroptosis share ROS storms and often occur synergistically. Since ROS simultaneously drives lipid peroxidation and activates receptor-interacting protein kinase 1 (RIPK1), these pathways serve as parallel and complementary clearance mechanisms in pathologies such as acute kidney injury [202]. There is a stronger link between ferroptosis and autophagy (a lysosomal-dependent process). As noted, selective autophagy, particularly NCOA4-mediated ferritinophagy, mobilizes the labile iron pool via ferritin degradation, thereby potently driving ferroptotic execution. Similarly, lipophagy provides the essential lipid substrates for peroxidation, positioning autophagic flux as a critical rheostat for ferroptotic sensitivity [203]. Moreover, ferroptosis and pyroptosis establish a “metabolic-to-immunogenic death” cascade. Damage-associated molecular patterns (DAMPs) released by ferroptotic cells activate the NACHT, NLRP3 inflammasome, triggering pyroptosis and the subsequent release of pro-inflammatory cytokines, which amplifies tissue damage or anti-tumor immunity [204,205]. Additionally, ferroptosis intersects with emerging cuproptosis through mitochondrial metabolic stress and metal toxicity [206], and with paraptosis via disrupted redox and ionic homeostasis—both converging on mitochondrial dysfunction as a shared execution node [207]. In conclusion, ferroptosis integrates with other RCD programs to form an interconnected decision-making network, orchestrated by shared ROS signaling, universal molecular hubs, and dynamic transition mechanisms. Given that most diseases involve a complex interplay of multiple RCD subroutines, the perceived “dominance” of ferroptosis may be confined to specific spatiotemporal windows or cellular subpopulations. Moreover, current evidence relies heavily on the rescue effects of inhibitors like Ferrostatin-1, which may exert non-specific effects on other ROS-dependent pathways, necessitating careful interpretation of the “dominance”. Ultimately, while characterizing a single modality as dominant may oversimplify the dynamic death network, targeting ferroptosis remains an indisputably promising therapeutic approach.

However, many mechanistic details remain incompletely understood. Emerging evidence suggests that lipid peroxides initially accumulate in organelles, such as the endoplasmic reticulum, before propagating to the plasma membrane [18,208], where they activate mechanosensitive ion channels including Piezo1 and transient receptor potential (TRP) channels [209]. This channel activation disrupts ion homeostasis, ultimately leading to membrane rupture. However, how lipid peroxidation precisely modulates membrane biophysical properties, and why interventions such as polyethylene glycol only delay but not prevent rupture [210], indicate the involvement of multiple contributing factors. Future studies could employ super-resolution imaging to visualize the dynamics of membrane nanopore formation and explore specific inhibitors targeting mechanosensitive channels to postpone membrane disintegration.

Furthermore, the compartmentalized regulation of lipid metabolism—such as the synthesis and trafficking of phosphatidylethanolamine—and the precise subcellular localization of key enzymes like ACSL4 remain unclear [211]. Interorganellar communication via membrane contact sites may facilitate the spread of lipid peroxidation, although the underlying mechanisms are not yet defined. Mitochondria and lipid droplets appear to play context-dependent roles in ferroptosis, with their contributions varying across cell types and microenvironments. Establishing conditional models that account for the functional heterogeneity of these organelles may help resolve existing controversies.

Iron metabolism represents another critical layer of regulation. The contributions of various iron sources—including transferrin-bound iron, iron-sulfur clusters, and heme—as well as iron release mechanisms such as ferritinophagy and quinone-mediated pathways [212], are not fully elucidated, particularly in mammalian systems [213]. In physiological and pathological contexts, ferroptosis is involved in development, aging, immune surveillance, and infection. How pathogens exploit ferroptosis to promote their survival, and how hosts counteract this process, remain important areas of investigation. Developing combination therapies involving ferroptosis inhibitors together with antibiotics or immunotherapies requires careful evaluation of potential benefits and risks. Notably, ferroptosis exerts dual effects in immune regulation: it can enhance antitumor immunity but may also suppress immune tolerance [214]. Its precise modulation poses a challenge for cancer therapy. Current research often focuses on how immune cells such as T cells and macrophages regulate ferroptosis sensitivity via cytokines like IFNγ [215,216,217,218]. Therefore, establishing reliable in vivo models to dissect the complex immune microenvironment is essential. Moreover, given that ferroptosis involves intercellular propagation and feedback mechanisms, organoid models may serve as an ideal system for further exploration.

Although ferroptosis initiation is of fundamental importance, accumulating evidence suggests that its onset is governed by multiple, context-dependent mechanisms, potentially involving coordinated redox reactions, iron handling, lipid metabolic remodeling, and upstream signaling pathways such as kinase cascades [173]. Because these early triggering events remain incompletely defined and are actively debated, they are not discussed in detail here. Instead, this Review focuses on the downstream amplification, execution, and propagation of ferroptosis damage, while highlighting ferroptosis initiation as a critical and open area for future investigation.

## Data Availability

No new data were created or analyzed in this study. Data sharing is not applicable to this article.

## Abbreviations

The following abbreviations are used in this manuscript:

7-DHC	7-dehydrocholesterol
ACSL4	Acyl-CoA synthetase long-chain family member 4
AKR1C1	Aldo-keto reductase family 1 member C1
ALOX	Arachidonate lipoxygenase
ATF	Activating transcription factor
ATM	Ataxia-telangiectasia mutated
BH4	Tetrahydrobiopterin
CDKN1A	Cyclin-dependent kinase inhibitor 1A
CoQ10H_2_	Reduced coenzyme Q10
CoQH_2_	Ubiquinol
CRE	cAMP response element
CYB5R1	NADH-cytochrome b5 reductase
DAMP	Damage-associated molecular pattern
DEHP	Di(2-ethylhexyl) phthalate
DGAT1	Diacylglycerol acyltransferase 1
DMT1	Divalent metal transporter 1
EMCS	ER-mitochondrial contact site
ER	Endoplasmic reticulum
FSP1	Ferroptosis suppressor protein 1
FTH1	Ferritin heavy chain 1
GCH1	GTP cyclohydroxylase 1
GPX4	Glutathione peroxidase 4
GSDME	Gasdermin E
HIF-1α	Hypoxia inducible factor-1 alphaMultidisciplinary
HO-1	Heme oxygenase 1
HSPA5	Heat shock protein family A member 5
iPLA2β	Calcium-independent phospholipase A2β
IRI	Ischemia–reperfusion injury
LCN2	Lipocalin 2
LD	Lipid droplet
LPCAT3	Lysophosphatidylcholine acyltransferase 3
MAPK	Mitogen-activated protein kinase
MBOAT1/2	Membrane-bound O-acyltransferase domain-containing 1 and 2
MFN2	Mitofusin 2
MOMP	Mitochondrial outer membrane permeabilization
mTOR	Mechanistic target of rapamycin
MUFA	Monounsaturated fatty acid
NCOA4	Nuclear receptor coactivator 4
NDRV	Novel duck reovirus
NF-κB	Nuclear factor of κ-light chain of enhancer-activated B cells family
NLRP3	The NACHT, LRR and PYD domains-containing protein 3
NOX	NAD(P)H oxidase
NRF	Necrosis-related factor
NRF2	Nuclear factor erythroid 2-related factor 2
NUPR1	Nuclear protein 1
OPA1	Optic atrophy 1
PE-PUFA	PUFA-containing phosphatidylethanolamine
PLA2	Phospholipase A2
PLTP	Phospholipid transfer protein
PMAIP1	Phorbol-12-myristate-13-acetate-induced protein 1
POR	NADPH-cytochrome P450 reductase
PRDX6	Peroxiredoxin 6
PUFA	Polyunsaturated fatty acid
PUMA	p53 upregulated modulator of apoptosis
RCD	Regulated cell death
RIPK1	Receptor-interacting protein kinase 1
ROS	Reactive oxygen species
RSS	Reactive sulfur species
RTA	Radical-trapping antioxidant
SCD1	Stearoyl-CoA desaturase-1
SLC7A11	Solute carrier family 7 member 11
STAT3	Signal transducer and activator of transcription 3
TFEB	The transcription factor EB
TfR1	Transferrin receptor 1
TRP	Transient receptor potential
VDAC1	Voltage-dependent anion channel 1

## References

[1] Stockwell B.R., Friedmann Angeli J.P., Bayir H., Bush A.I., Conrad M., Dixon S.J., Fulda S., Gascón S., Hatzios S.K., Kagan V.E. (2017). Ferroptosis: A Regulated Cell Death Nexus Linking Metabolism, Redox Biology, and Disease. Cell.

[2] Dixon S.J., Lemberg K.M., Lamprecht M.R., Skouta R., Zaitsev E.M., Gleason C.E., Patel D.N., Bauer A.J., Cantley A.M., Yang W.S. (2012). Ferroptosis: An iron-dependent form of nonapoptotic cell death. Cell.

[3] Dixon S.J., Olzmann J.A. (2024). The cell biology of ferroptosis. Nat. Rev. Mol. Cell Biol..

[4] Riegman M., Bradbury M.S., Overholtzer M. (2019). Population Dynamics in Cell Death: Mechanisms of Propagation. Trends Cancer.

[5] Lin Y., Xu W., Hou Y., Wang S., Zhang H., Ran M., Huang Y., Wang Y., Yang G. (2022). The multifaceted role of ferroptosis in kidney diseases. Chem. Biol. Interact..

[6] Zhou Y., Lin W., Rao T., Zheng J., Zhang T., Zhang M., Lin Z. (2022). Ferroptosis and Its Potential Role in the Nervous System Diseases. J. Inflamm. Res..

[7] Shrivastav D., Mishra J., Sharma V.K., Singh S., Khan M.I., Alsanie S.A., Ashfaq F., Beg M.M.A. (2025). Biochemical and Physiological Response During Oxidative Stress: A Cross-Species Perspective. Rejuvenation Res..

[8] Huang Q., Ru Y., Luo Y., Luo X., Liu D., Ma Y., Zhou X., Linghu M., Xu W., Gao F. (2024). Identification of a targeted ACSL4 inhibitor to treat ferroptosis-related diseases. Sci. Adv..

[9] Shui S., Zhao Z., Wang H., Conrad M., Liu G. (2021). Non-enzymatic lipid peroxidation initiated by photodynamic therapy drives a distinct ferroptosis-like cell death pathway. Redox Biol..

[10] Chen D., Chu B., Yang X., Liu Z., Jin Y., Kon N., Rabadan R., Jiang X., Stockwell B.R., Gu W. (2021). iPLA2β-mediated lipid detoxification controls p53-driven ferroptosis independent of GPX4. Nat. Commun..

[11] Jiang L., Kon N., Li T., Wang S.-J., Su T., Hibshoosh H., Baer R., Gu W. (2015). Ferroptosis as a p53-mediated activity during tumour suppression. Nature.

[12] Li Y., Ran Q., Duan Q., Jin J., Wang Y., Yu L., Wang C., Zhu Z., Chen X., Weng L. (2024). 7-Dehydrocholesterol dictates ferroptosis sensitivity. Nature.

[13] Freitas F.P., Alborzinia H., Dos Santos A.F., Nepachalovich P., Pedrera L., Zilka O., Inague A., Klein C., Aroua N., Kaushal K. (2024). 7-Dehydrocholesterol is an endogenous suppressor of ferroptosis. Nature.

[14] Yang W.S., Kim K.J., Gaschler M.M., Patel M., Shchepinov M.S., Stockwell B.R. (2016). Peroxidation of polyunsaturated fatty acids by lipoxygenases drives ferroptosis. Proc. Natl. Acad. Sci. USA.

[15] Kagan V.E., Mao G., Qu F., Angeli J.P.F., Doll S., Croix C.S., Dar H.H., Liu B., Tyurin V.A., Ritov V.B. (2017). Oxidized arachidonic and adrenic PEs navigate cells to ferroptosis. Nat. Chem. Biol..

[16] Anthonymuthu T.S., Tyurina Y.Y., Sun W.-Y., Mikulska-Ruminska K., Shrivastava I.H., Tyurin V.A., Cinemre F.B., Dar H.H., Van Demark A.P., Holman T.R. (2021). Resolving the paradox of ferroptotic cell death: Ferrostatin-1 binds to 15LOX/PEBP1 complex, suppresses generation of peroxidized ETE-PE, and protects against ferroptosis. Redox Biol..

[17] Doll S., Proneth B., Tyurina Y.Y., Panzilius E., Kobayashi S., Ingold I., Irmler M., Beckers J., Aichler M., Walch A. (2017). ACSL4 dictates ferroptosis sensitivity by shaping cellular lipid composition. Nat. Chem. Biol..

[18] Magtanong L., Mueller G.D., Williams K.J., Billmann M., Chan K., Armenta D.A., Pope L.E., Moffat J., Boone C., Myers C.L. (2022). Context-dependent regulation of ferroptosis sensitivity. Cell Chem. Biol..

[19] Chu B., Kon N., Chen D., Li T., Liu T., Jiang L., Song S., Tavana O., Gu W. (2019). ALOX12 is required for p53-mediated tumour suppression through a distinct ferroptosis pathway. Nat. Cell Biol..

[20] Correia J.H., Rodrigues J.A., Pimenta S., Dong T., Yang Z. (2021). Photodynamic Therapy Review: Principles, Photosensitizers, Applications, and Future Directions. Pharmaceutics.

[21] Qiu B., Zandkarimi F., Bezjian C.T., Reznik E., Soni R.K., Gu W., Jiang X., Stockwell B.R. (2024). Phospholipids with two polyunsaturated fatty acyl tails promote ferroptosis. Cell.

[22] Lorent J.H., Levental K.R., Ganesan L., Rivera-Longsworth G., Sezgin E., Doktorova M., Lyman E., Levental I. (2020). Plasma membranes are asymmetric in lipid unsaturation, packing and protein shape. Nat. Chem. Biol..

[23] Zou Y., Henry W.S., Ricq E.L., Graham E.T., Phadnis V.V., Maretich P., Paradkar S., Boehnke N., Deik A.A., Reinhardt F. (2020). Plasticity of ether lipids promotes ferroptosis susceptibility and evasion. Nature.

[24] Magtanong L., Ko P.-J., To M., Cao J.Y., Forcina G.C., Tarangelo A., Ward C.C., Cho K., Patti G.J., Nomura D.K. (2019). Exogenous Monounsaturated Fatty Acids Promote a Ferroptosis-Resistant Cell State. Cell Chem. Biol..

[25] Smolková K., Gotvaldová K. (2025). Fatty Acid Trafficking Between Lipid Droplets and Mitochondria: An Emerging Perspective. Int. J. Biol. Sci..

[26] Jarc E., Kump A., Malavašič P., Eichmann T.O., Zimmermann R., Petan T. (2018). Lipid droplets induced by secreted phospholipase A2 and unsaturated fatty acids protect breast cancer cells from nutrient and lipotoxic stress. Biochim. Biophys. Acta Mol. Cell Biol. Lipids.

[27] Danielli M., Perne L., Jarc Jovičić E., Petan T. (2023). Lipid droplets and polyunsaturated fatty acid trafficking: Balancing life and death. Front. Cell Dev. Biol..

[28] Ferrada L., Barahona M.J., Vera M., Stockwell B.R., Nualart F. (2023). Dehydroascorbic acid sensitizes cancer cells to system xc- inhibition-induced ferroptosis by promoting lipid droplet peroxidation. Cell Death Dis..

[29] Conrad M., Pratt D.A. (2019). The chemical basis of ferroptosis. Nat. Chem. Biol..

[30] Li Z., Lange M., Dixon S.J., Olzmann J.A. (2024). Lipid Quality Control and Ferroptosis: From Concept to Mechanism. Annu. Rev. Biochem..

[31] Yan B., Ai Y., Sun Q., Ma Y., Cao Y., Wang J., Zhang Z., Wang X. (2021). Membrane Damage during Ferroptosis Is Caused by Oxidation of Phospholipids Catalyzed by the Oxidoreductases POR and CYB5R1. Mol. Cell.

[32] Vermot A., Petit-Härtlein I., Smith S.M.E., Fieschi F. (2021). NADPH Oxidases (NOX): An Overview from Discovery, Molecular Mechanisms to Physiology and Pathology. Antioxidants.

[33] Song S., Su Z., Kon N., Chu B., Li H., Jiang X., Luo J., Stockwell B.R., Gu W. (2023). ALOX5-mediated ferroptosis acts as a distinct cell death pathway upon oxidative stress in Huntington’s disease. Genes Dev..

[34] Feng Y., Wang X., Li P., Shi X., Prokosch V., Liu H. (2025). Exogenous hydrogen sulfide and NOX2 inhibition mitigate ferroptosis in pressure-induced retinal ganglion cell damage. Biochim. Biophys. Acta Mol. Basis Dis..

[35] Li K., Wang M., Huang Z.-H., Wang M., Sun W.-Y., Kurihara H., Huang R.-T., Wang R., Huang F., Liang L. (2023). ALOX5 inhibition protects against dopaminergic neurons undergoing ferroptosis. Pharmacol. Res..

[36] Karuppagounder S.S., Alin L., Chen Y., Brand D., Bourassa M.W., Dietrich K., Wilkinson C.M., Nadeau C.A., Kumar A., Perry S. (2018). N-acetylcysteine targets 5 lipoxygenase-derived, toxic lipids and can synergize with prostaglandin E2 to inhibit ferroptosis and improve outcomes following hemorrhagic stroke in mice. Ann. Neurol..

[37] Mao X., Wang X., Jin M., Li Q., Jia J., Li M., Zhou H., Liu Z., Jin W., Zhao Y. (2022). Critical involvement of lysyl oxidase in seizure-induced neuronal damage through ERK-Alox5-dependent ferroptosis and its therapeutic implications. Acta Pharm. Sin. B.

[38] Yang W.-H., Huang Z., Wu J., Ding C.-K.C., Murphy S.K., Chi J.-T. (2020). A TAZ-ANGPTL4-NOX2 Axis Regulates Ferroptotic Cell Death and Chemoresistance in Epithelial Ovarian Cancer. Mol. Cancer Res..

[39] Yang W.-H., Ding C.-K.C., Sun T., Rupprecht G., Lin C.-C., Hsu D., Chi J.-T. (2019). The Hippo Pathway Effector TAZ Regulates Ferroptosis in Renal Cell Carcinoma. Cell Rep..

[40] Shah R., Shchepinov M.S., Pratt D.A. (2018). Resolving the Role of Lipoxygenases in the Initiation and Execution of Ferroptosis. ACS Cent. Sci..

[41] Ai Y., Yan B., Wang X. (2021). The oxidoreductases POR and CYB5R1 catalyze lipid peroxidation to execute ferroptosis. Mol. Cell Oncol..

[42] Galy B., Conrad M., Muckenthaler M. (2024). Mechanisms controlling cellular and systemic iron homeostasis. Nat. Rev. Mol. Cell Biol..

[43] Zhang W., Liu Y., Liao Y., Zhu C., Zou Z. (2024). GPX4, ferroptosis, and diseases. Biomed. Pharmacother..

[44] Yang W.S., SriRamaratnam R., Welsch M.E., Shimada K., Skouta R., Viswanathan V.S., Cheah J.H., Clemons P.A., Shamji A.F., Clish C.B. (2014). Regulation of ferroptotic cancer cell death by GPX4. Cell.

[45] Bersuker K., Hendricks J.M., Li Z., Magtanong L., Ford B., Tang P.H., Roberts M.A., Tong B., Maimone T.J., Zoncu R. (2019). The CoQ oxidoreductase FSP1 acts parallel to GPX4 to inhibit ferroptosis. Nature.

[46] Fisher A.B. (2011). Peroxiredoxin 6: A bifunctional enzyme with glutathione peroxidase and phospholipase A_2_ activities. Antioxid. Redox Signal..

[47] Kraft V.A.N., Bezjian C.T., Pfeiffer S., Ringelstetter L., Müller C., Zandkarimi F., Merl-Pham J., Bao X., Anastasov N., Kössl J. (2020). GTP Cyclohydrolase 1/Tetrahydrobiopterin Counteract Ferroptosis through Lipid Remodeling. ACS Cent. Sci..

[48] Liu C., Zhang C., Wu H., Zhao Z., Wang Z., Zhang X., Yang J., Yu W., Lian Z., Gao M. (2025). The AKR1C1-CYP1B1-cAMP signaling axis controls tumorigenicity and ferroptosis susceptibility of extrahepatic cholangiocarcinoma. Cell Death Differ..

[49] Zhang R., Kroemer G., Tang D. (2024). Lipid-derived radical-trapping antioxidants suppress ferroptosis. Life Metab..

[50] Chen M., Ghelfi M., Poon J.-F., Jeon N., Boccalon N., Rubsamen M., Valentino S., Mehta V., Stamper M., Tariq H. (2025). Antioxidant-independent activities of alpha-tocopherol. J. Biol. Chem..

[51] Doll S., Freitas F.P., Shah R., Aldrovandi M., Da Silva M.C., Ingold I., Goya Grocin A., Da Xavier Silva T.N., Panzilius E., Scheel C.H. (2019). FSP1 is a glutathione-independent ferroptosis suppressor. Nature.

[52] Li W., Liang L., Liu S., Yi H., Zhou Y. (2023). FSP1: A key regulator of ferroptosis. Trends Mol. Med..

[53] Zhang M., Chen X., Zhang Y. (2024). Mechanisms of Vitamins Inhibiting Ferroptosis. Antioxidants.

[54] Mishima E., Ito J., Wu Z., Nakamura T., Wahida A., Doll S., Tonnus W., Nepachalovich P., Eggenhofer E., Aldrovandi M. (2022). A non-canonical vitamin K cycle is a potent ferroptosis suppressor. Nature.

[55] Jakaria M., Belaidi A.A., Bush A.I., Ayton S. (2023). Vitamin A metabolites inhibit ferroptosis. Biomed. Pharmacother..

[56] Li J., Cao Y., Xu J., Li J., Lv C., Gao Q., Zhang C., Jin C., Wang R., Jiao R. (2023). Vitamin D Improves Cognitive Impairment and Alleviates Ferroptosis via the Nrf2 Signaling Pathway in Aging Mice. Int. J. Mol. Sci..

[57] Miao Y., Jiang Z., Song H., Zhang Y., Chen H., Liu W., Wei X., Li L., Li W., Li X. (2024). Vitamin D supplementation alleviates high fat diet-induced metabolic associated fatty liver disease by inhibiting ferroptosis pathway. Eur. J. Nutr..

[58] Tschuck J., Padmanabhan Nair V., Galhoz A., Zaratiegui C., Tai H.-M., Ciceri G., Rothenaigner I., Tchieu J., Stockwell B.R., Studer L. (2024). Suppression of ferroptosis by vitamin A or radical-trapping antioxidants is essential for neuronal development. Nat. Commun..

[59] Sun G., Liu C., Lu Z., Zhang J., Cao H., Huang T., Dai M., Liu H., Feng T., Tang W. (2024). Metabolomics reveals ascorbic acid inhibits ferroptosis in hepatocytes and boosts the effectiveness of anti-PD1 immunotherapy in hepatocellular carcinoma. Cancer Cell Int..

[60] Chen J., Fu Y., Weng S., He J., Dong C. (2025). Vitamin C Inhibits Scale Drop Disease Virus Infectivity by Targeting Nrf2 to Reduce Ferroptosis. Antioxidants.

[61] Iciek M., Bilska-Wilkosz A., Kozdrowicki M., Górny M. (2022). Reactive sulfur species and their significance in health and disease. Biosci. Rep..

[62] Soula M., Weber R.A., Zilka O., Alwaseem H., La K., Yen F., Molina H., Garcia-Bermudez J., Pratt D.A., Birsoy K. (2020). Metabolic determinants of cancer cell sensitivity to canonical ferroptosis inducers. Nat. Chem. Biol..

[63] Buettner G.R. (1993). The pecking order of free radicals and antioxidants: Lipid peroxidation, alpha-tocopherol, and ascorbate. Arch. Biochem. Biophys..

[64] Fukuto J.M. (2022). The Biological/Physiological Utility of Hydropersulfides (RSSH) and Related Species: What Is Old Is New Again. Antioxid. Redox Signal..

[65] Chen Y., Jiang Z., Li X. (2024). New insights into crosstalk between Nrf2 pathway and ferroptosis in lung disease. Cell Death Dis..

[66] Volyar A., Bretsko M., Khalilov S., Akimova Y. (2025). Self-healing and self-matching effects in astigmatic structured beams as a basis for measuring orbital Stokes parameters. Appl. Opt..

[67] Nagini S., Kallamadi P.R., Tanagala K.K.K., Reddy G.B. (2024). Aldo-keto reductases: Role in cancer development and theranostics. Oncol. Res..

[68] Duan C., Jiao D., Wang H., Wu Q., Men W., Yan H., Li C. (2022). Activation of the PPARγ Prevents Ferroptosis-Induced Neuronal Loss in Response to Intracerebral Hemorrhage Through Synergistic Actions With the Nrf2. Front. Pharmacol..

[69] Anandhan A., Dodson M., Shakya A., Chen J., Liu P., Wei Y., Tan H., Wang Q., Jiang Z., Yang K. (2023). NRF2 controls iron homeostasis and ferroptosis through HERC2 and VAMP8. Sci. Adv..

[70] Singh A., Venkannagari S., Oh K.H., Zhang Y.-Q., Rohde J.M., Liu L., Nimmagadda S., Sudini K., Brimacombe K.R., Gajghate S. (2016). Small Molecule Inhibitor of NRF2 Selectively Intervenes Therapeutic Resistance in KEAP1-Deficient NSCLC Tumors. ACS Chem. Biol..

[71] Yan L., Hu H., Feng L., Li Z., Zheng C., Zhang J., Yin X., Li B. (2024). ML385 promotes ferroptosis and radiotherapy sensitivity by inhibiting the NRF2-SLC7A11 pathway in esophageal squamous cell carcinoma. Med. Oncol..

[72] Yuan J., Huang W., Lin M., Sun S., Zhong F., Ye L., Yin H., Ou X., Zeng Z. (2025). ML385 increases ferroptosis via inhibiting Nrf2/HO-1 pathway to enhances the sensitivity of MCF-7 TAMR to tamoxifen. Naunyn Schmiedebergs. Arch. Pharmacol..

[73] Shen J., Chen S., Li X., Wu L., Mao X., Jiang J., Zhu D. (2024). Salidroside Mediated the Nrf2/GPX4 Pathway to Attenuates Ferroptosis in Parkinson’s Disease. Neurochem. Res..

[74] Liu P., Anandhan A., Chen J., Shakya A., Dodson M., Ooi A., Chapman E., White E., Garcia J.G., Zhang D.D. (2023). Decreased autophagosome biogenesis, reduced NRF2, and enhanced ferroptotic cell death are underlying molecular mechanisms of non-alcoholic fatty liver disease. Redox Biol..

[75] Zhang X., Li Z., Zhang X., Yuan Z., Zhang L., Miao P. (2024). ATF family members as therapeutic targets in cancer: From mechanisms to pharmacological interventions. Pharmacol. Res..

[76] Xu X., Li Y., Wu Y., Wang M., Lu Y., Fang Z., Wang H., Li Y. (2023). Increased ATF2 expression predicts poor prognosis and inhibits sorafenib-induced ferroptosis in gastric cancer. Redox Biol..

[77] Shao C.-J., Zhou H.-L., Gao X.-Z., Xu C.-F. (2023). Downregulation of miR-221-3p promotes the ferroptosis in gastric cancer cells via upregulation of ATF3 to mediate the transcription inhibition of GPX4 and HRD1. Transl. Oncol..

[78] Wang L., Liu Y., Du T., Yang H., Lei L., Guo M., Ding H.-F., Zhang J., Wang H., Chen X. (2020). ATF3 promotes erastin-induced ferroptosis by suppressing system Xc. Cell Death Differ..

[79] Kim K.-H., Jeong J.-Y., Surh Y.-J., Kim K.-W. (2010). Expression of stress-response ATF3 is mediated by Nrf2 in astrocytes. Nucleic Acids Res..

[80] Zhu S., Zhang Q., Sun X., Zeh H.J., Lotze M.T., Kang R., Tang D. (2017). HSPA5 Regulates Ferroptotic Cell Death in Cancer Cells. Cancer Res..

[81] He F., Zhang P., Liu J., Wang R., Kaufman R.J., Yaden B.C., Karin M. (2023). ATF4 suppresses hepatocarcinogenesis by inducing SLC7A11 (xCT) to block stress-related ferroptosis. J. Hepatol..

[82] Liu J., Song X., Kuang F., Zhang Q., Xie Y., Kang R., Kroemer G., Tang D. (2021). NUPR1 is a critical repressor of ferroptosis. Nat. Commun..

[83] Gao R., Kalathur R.K.R., Coto-Llerena M., Ercan C., Buechel D., Shuang S., Piscuoglio S., Dill M.T., Camargo F.D., Christofori G. (2021). YAP/TAZ and ATF4 drive resistance to Sorafenib in hepatocellular carcinoma by preventing ferroptosis. EMBO Mol. Med..

[84] Kreß J.K.C., Jessen C., Hufnagel A., Schmitz W., Da Xavier Silva T.N., Ferreira Dos Santos A., Mosteo L., Goding C.R., Friedmann Angeli J.P., Meierjohann S. (2023). The integrated stress response effector ATF4 is an obligatory metabolic activator of NRF2. Cell Rep..

[85] Chen Y., Fang Z.-M., Yi X., Wei X., Jiang D.-S. (2023). The interaction between ferroptosis and inflammatory signaling pathways. Cell Death Dis..

[86] Wang Y.-F., Feng J.-Y., Zhao L.-N., Zhao M., Wei X.-F., Geng Y., Yuan H.-F., Hou C.-Y., Zhang H.-H., Wang G.-W. (2023). Aspirin triggers ferroptosis in hepatocellular carcinoma cells through restricting NF-κB p65-activated SLC7A11 transcription. Acta Pharmacol. Sin..

[87] Yao F., Deng Y., Zhao Y., Mei Y., Zhang Y., Liu X., Martinez C., Su X., Rosato R.R., Teng H. (2021). A targetable LIFR-NF-κB-LCN2 axis controls liver tumorigenesis and vulnerability to ferroptosis. Nat. Commun..

[88] Schmitt A., Xu W., Bucher P., Grimm M., Konantz M., Horn H., Zapukhlyak M., Berning P., Brändle M., Jarboui M.-A. (2021). Dimethyl fumarate induces ferroptosis and impairs NF-κB/STAT3 signaling in DLBCL. Blood.

[89] Ding Z., Pan Y., Shang T., Jiang T., Lin Y., Yang C., Pang S., Cui X., Wang Y., Feng X.F. (2023). URI alleviates tyrosine kinase inhibitors-induced ferroptosis by reprogramming lipid metabolism in p53 wild-type liver cancers. Nat. Commun..

[90] Rodencal J., Kim N., He A., Li V.L., Lange M., He J., Tarangelo A., Schafer Z.T., Olzmann J.A., Long J.Z. (2024). Sensitization of cancer cells to ferroptosis coincident with cell cycle arrest. Cell Chem. Biol..

[91] Ye S., Xu M., Zhu T., Chen J., Shi S., Jiang H., Zheng Q., Liao Q., Ding X., Xi Y. (2021). Cytoglobin promotes sensitivity to ferroptosis by regulating p53-YAP1 axis in colon cancer cells. J. Cell Mol. Med..

[92] Gnanapradeepan K., Indeglia A., Stieg D.C., Clarke N., Shao C., Dougherty J.F., Murali N., Murphy M.E. (2022). PLTP is a p53 target gene with roles in cancer growth suppression and ferroptosis. J. Biol. Chem..

[93] Tang D., Chen X., Kang R., Kroemer G. (2021). Ferroptosis: Molecular mechanisms and health implications. Cell Res..

[94] Aydin E., Johansson J., Nazir F.H., Hellstrand K., Martner A. (2017). Role of NOX2-Derived Reactive Oxygen Species in NK Cell-Mediated Control of Murine Melanoma Metastasis. Cancer Immunol. Res..

[95] Ji H., Wang W., Li X., Han X., Zhang X., Wang J., Liu C., Huang L., Gao W. (2022). p53: A double-edged sword in tumor ferroptosis. Pharmacol. Res..

[96] Ciamporcero E., Daga M., Pizzimenti S., Roetto A., Dianzani C., Compagnone A., Palmieri A., Ullio C., Cangemi L., Pili R. (2018). Crosstalk between Nrf2 and YAP contributes to maintaining the antioxidant potential and chemoresistance in bladder cancer. Free Radic. Biol. Med..

[97] Dai J.-Z., Hsu W.-J., Lin M.-H., Shueng P.-W., Lee C.-C., Yang C.-C., Lin C.-W. (2025). YAP-mediated DDX3X confers resistance to ferroptosis in breast cancer cells by reducing lipid peroxidation. Free Radic. Biol. Med..

[98] Cao G., Yin S., Ma J., Lu Y., Song R., Wu Z., Liu C., Liu J., Wu P., Sun R. (2024). YAP promotes the healing of ischemic wounds by reducing ferroptosis in skin fibroblasts through inhibition of ferritinophagy. Heliyon.

[99] Mo M., Pan L., Deng L., Liang M., Xia N., Liang Y. (2024). Iron Overload Induces Hepatic Ferroptosis and Insulin Resistance by Inhibiting the Jak2/stat3/slc7a11 Signaling Pathway. Cell Biochem. Biophys..

[100] Poindessous V., Lazareth H., Crambert G., Cheval L., Sampaio J.L., Pallet N. (2024). STAT3 drives the expression of ACSL4 in acute kidney injury. iScience.

[101] Chen L., Ma Y., Ma X., Liu L., Jv X., Li A., Shen Q., Jia W., Qu L., Shi L. (2023). TFEB regulates cellular labile iron and prevents ferroptosis in a TfR1-dependent manner. Free Radic. Biol. Med..

[102] Sun X.-J., Xiao S.-J., Ma W.-Q., Jin H., Ren L.-Q., Yao Y.-Y., Chen Z.-D., Li X.-X., Chen T., Liu N.-F. (2025). Activation of TFEB protects against diabetic vascular calcification by improving autophagic flux and activating Nrf2 antioxidant system. Am. J. Physiol. Endocrinol. Metab..

[103] Fang W., Song X., Li H., Meng F., Lv T., Huang J., Ji X., Lv J., Cai Z., Wang Z. (2024). Wnt/β-catenin signaling inhibits oxidative stress-induced ferroptosis to improve interstitial cystitis/bladder pain syndrome by reducing NF-κB. Biochim. Biophys. Acta Mol. Cell Res..

[104] Linkermann A., Skouta R., Himmerkus N., Mulay S.R., Dewitz C., De Zen F., Prokai A., Zuchtriegel G., Krombach F., Welz P.-S. (2014). Synchronized renal tubular cell death involves ferroptosis. Proc. Natl. Acad. Sci. USA.

[105] Roth T.L., Nayak D., Atanasijevic T., Koretsky A.P., Latour L.L., McGavern D.B. (2014). Transcranial amelioration of inflammation and cell death after brain injury. Nature.

[106] Li Y., Feng D., Wang Z., Zhao Y., Sun R., Tian D., Liu D., Zhang F., Ning S., Yao J. (2019). Ischemia-induced ACSL4 activation contributes to ferroptosis-mediated tissue injury in intestinal ischemia/reperfusion. Cell Death Differ..

[107] Tang Z., Zhao P., Wang H., Liu Y., Bu W. (2021). Biomedicine Meets Fenton Chemistry. Chem. Rev..

[108] Wenzel S.E., Tyurina Y.Y., Zhao J., St Croix C.M., Dar H.H., Mao G., Tyurin V.A., Anthonymuthu T.S., Kapralov A.A., Amoscato A.A. (2017). PEBP1 Wardens Ferroptosis by Enabling Lipoxygenase Generation of Lipid Death Signals. Cell.

[109] Marnett L.J., Wilcox A.L. (1995). The chemistry of lipid alkoxyl radicals and their role in metal-amplified lipid peroxidation. Biochem. Soc. Symp..

[110] Park E., Chung S.W. (2019). ROS-mediated autophagy increases intracellular iron levels and ferroptosis by ferritin and transferrin receptor regulation. Cell Death Dis..

[111] Xie X., Zhang Y., Wang Z., Wang S., Jiang X., Cui H., Zhou T., He Z., Feng H., Guo Q. (2021). ATM at the crossroads of reactive oxygen species and autophagy. Int. J. Biol. Sci..

[112] Chen W., Yang W., Zhang C., Liu T., Zhu J., Wang H., Li T., Jin A., Ding L., Xian J. (2022). Modulation of the p38 MAPK Pathway by Anisomycin Promotes Ferroptosis of Hepatocellular Carcinoma through Phosphorylation of H3S10. Oxid. Med. Cell Longev..

[113] Liu Y., Wang Y., Liu J., Kang R., Tang D. (2021). Interplay between MTOR and GPX4 signaling modulates autophagy-dependent ferroptotic cancer cell death. Cancer Gene Ther..

[114] Wu H., Liu Q., Shan X., Gao W., Chen Q. (2023). ATM orchestrates ferritinophagy and ferroptosis by phosphorylating NCOA4. Autophagy.

[115] Bai Y., Meng L., Han L., Jia Y., Zhao Y., Gao H., Kang R., Wang X., Tang D., Dai E. (2019). Lipid storage and lipophagy regulates ferroptosis. Biochem. Biophys. Res. Commun..

[116] Schroeder B., Schulze R.J., Weller S.G., Sletten A.C., Casey C.A., McNiven M.A. (2015). The small GTPase Rab7 as a central regulator of hepatocellular lipophagy. Hepatology.

[117] Gao M., Monian P., Pan Q., Zhang W., Xiang J., Jiang X. (2016). Ferroptosis is an autophagic cell death process. Cell Res..

[118] Sun Y., Berleth N., Wu W., Schlütermann D., Deitersen J., Stuhldreier F., Berning L., Friedrich A., Akgün S., Mendiburo M.J. (2021). Fin56-induced ferroptosis is supported by autophagy-mediated GPX4 degradation and functions synergistically with mTOR inhibition to kill bladder cancer cells. Cell Death Dis..

[119] Sassano M.L., Tyurina Y.Y., Diokmetzidou A., Vervoort E., Tyurin V.A., More S., La Rovere R., Giordano F., Bultynck G., Pavie B. (2025). Endoplasmic reticulum-mitochondria contacts are prime hotspots of phospholipid peroxidation driving ferroptosis. Nat. Cell Biol..

[120] Liu X., Hussain R., Mehmood K., Tang Z., Zhang H., Li Y. (2022). Mitochondrial-Endoplasmic Reticulum Communication-Mediated Oxidative Stress and Autophagy. Biomed Res. Int..

[121] Li M.-D., Fu L., Lv B.-B., Xiang Y., Xiang H.-X., Xu D.-X., Zhao H. (2022). Arsenic induces ferroptosis and acute lung injury through mtROS-mediated mitochondria-associated endoplasmic reticulum membrane dysfunction. Ecotoxicol. Environ. Saf..

[122] Liang F.G., Zandkarimi F., Lee J., Axelrod J.L., Pekson R., Yoon Y., Stockwell B.R., Kitsis R.N. (2024). OPA1 promotes ferroptosis by augmenting mitochondrial ROS and suppressing an integrated stress response. Mol. Cell.

[123] Kurz T., Terman A., Gustafsson B., Brunk U.T. (2008). Lysosomes in iron metabolism, ageing and apoptosis. Histochem. Cell Biol..

[124] Saimoto Y., Kusakabe D., Morimoto K., Matsuoka Y., Kozakura E., Kato N., Tsunematsu K., Umeno T., Kiyotani T., Matsumoto S. (2025). Lysosomal lipid peroxidation contributes to ferroptosis induction via lysosomal membrane permeabilization. Nat. Commun..

[125] Sheftel A.D., Zhang A.-S., Brown C., Shirihai O.S., Ponka P. (2007). Direct interorganellar transfer of iron from endosome to mitochondrion. Blood.

[126] Wong Y.C., Ysselstein D., Krainc D. (2018). Mitochondria-lysosome contacts regulate mitochondrial fission via RAB7 GTP hydrolysis. Nature.

[127] Wolff N.A., Garrick M.D., Zhao L., Garrick L.M., Ghio A.J., Thévenod F. (2018). A role for divalent metal transporter (DMT1) in mitochondrial uptake of iron and manganese. Sci. Rep..

[128] Qin S., Davern K., Wilson S.G., Chen K., Li A., Xu J. (2025). Lysosome–Iron–Mitochondria Axis in Osteoclasts: Iron as a Central Player. Research.

[129] Kiraly S., Stanley J., Eden E.R. (2025). Lysosome-Mitochondrial Crosstalk in Cellular Stress and Disease. Antioxidants.

[130] Wei S., Qiu T., Yao X., Wang N., Jiang L., Jia X., Tao Y., Wang Z., Pei P., Zhang J. (2020). Arsenic induces pancreatic dysfunction and ferroptosis via mitochondrial ROS-autophagy-lysosomal pathway. J. Hazard. Mater..

[131] Katikaneni A., Jelcic M., Gerlach G.F., Ma Y., Overholtzer M., Niethammer P. (2020). Lipid peroxidation regulates long-range wound detection through 5-lipoxygenase in zebrafish. Nat. Cell Biol..

[132] Riegman M., Sagie L., Galed C., Levin T., Steinberg N., Dixon S.J., Wiesner U., Bradbury M.S., Niethammer P., Zaritsky A. (2020). Ferroptosis occurs through an osmotic mechanism and propagates independently of cell rupture. Nat. Cell Biol..

[133] Roeck B.F., Lotfipour Nasudivar S., Vorndran M.R.H., Schueller L., Yapici F.I., Rübsam M., von Karstedt S., Niessen C.M., Garcia-Saez A.J. (2025). Ferroptosis spreads to neighboring cells via plasma membrane contacts. Nat. Commun..

[134] Co H.K.C., Wu C.-C., Lee Y.-C., Chen S.-H. (2024). Emergence of large-scale cell death through ferroptotic trigger waves. Nature.

[135] Hou M.-J., Wang P., Zhu B.T. (2023). Biochemical mechanism of erastin-induced ferroptotic cell death in neuronal cells. Acta Biochim. Biophys. Sin..

[136] Wang T., Tomas D., Perera N.D., Cuic B., Luikinga S., Viden A., Barton S.K., McLean C.A., Samson A.L., Southon A. (2022). Ferroptosis mediates selective motor neuron death in amyotrophic lateral sclerosis. Cell Death Differ..

[137] Skouta R., Dixon S.J., Wang J., Dunn D.E., Orman M., Shimada K., Rosenberg P.A., Lo D.C., Weinberg J.M., Linkermann A. (2014). Ferrostatins inhibit oxidative lipid damage and cell death in diverse disease models. J. Am. Chem. Soc..

[138] Zhang B., Chen K., Dai Y., Luo X., Xiong Z., Zhang W., Huang X., So K.-F., Zhang L. (2024). Human α-synuclein aggregation activates ferroptosis leading to parvalbumin interneuron degeneration and motor learning impairment. Commun. Biol..

[139] Mahoney-Sanchez L., Bouchaoui H., Boussaad I., Jonneaux A., Timmerman K., Berdeaux O., Ayton S., Krüger R., Duce J.A., Devos D. (2022). Alpha synuclein determines ferroptosis sensitivity in dopaminergic neurons via modulation of ether-phospholipid membrane composition. Cell Rep..

[140] Angelova P.R., Choi M.L., Berezhnov A.V., Horrocks M.H., Hughes C.D., De S., Rodrigues M., Yapom R., Little D., Dolt K.S. (2020). Alpha synuclein aggregation drives ferroptosis: An interplay of iron, calcium and lipid peroxidation. Cell Death Differ..

[141] Cozzi A., Orellana D.I., Santambrogio P., Rubio A., Cancellieri C., Giannelli S., Ripamonti M., Taverna S., Di Lullo G., Rovida E. (2019). Stem Cell Modeling of Neuroferritinopathy Reveals Iron as a Determinant of Senescence and Ferroptosis during Neuronal Aging. Stem Cell Rep..

[142] Lorenz S.M., Wahida A., Bostock M.J., Seibt T., Santos Dias Mourão A., Levkina A., Trümbach D., Soudy M., Emler D., Rothammer N. (2025). A fin-loop-like structure in GPX4 underlies neuroprotection from ferroptosis. Cell.

[143] Yan H.-F., Tuo Q.-Z., Yin Q.-Z., Lei P. (2020). The pathological role of ferroptosis in ischemia/reperfusion-related injury. Zool. Res..

[144] Fu H.-J., Zhou X.-Y., Qin D.-L., Qiao Q., Wang Q.-Z., Li S.-Y., Zhu Y.-F., Li Y.-P., Zhou J.-M., Cai H. (2025). Inhibition of Ferroptosis Delays Aging and Extends Healthspan Across Multiple Species. Adv. Sci..

[145] Mann J., Reznik E., Santer M., Fongheiser M.A., Smith N., Hirschhorn T., Zandkarimi F., Soni R.K., Dafré A.L., Miranda-Vizuete A. (2024). Ferroptosis inhibition by oleic acid mitigates iron-overload-induced injury. Cell Chem. Biol..

[146] Perez M.A., Magtanong L., Dixon S.J., Watts J.L. (2020). Dietary Lipids Induce Ferroptosis in Caenorhabditiselegans and Human Cancer Cells. Dev. Cell.

[147] Jiang Y.H., McGeachin R.B., Bailey C.A. (1994). alpha-tocopherol, beta-carotene, and retinol enrichment of chicken eggs. Poult. Sci..

[148] Chen Y., Hao Z., Lv Z., Ning Z., Guo Y., Yuan J. (2025). Comparative Effects of Organic and Nano-Selenium on Egg Quality and Antioxidant Capacity in Layer Hens. Foods.

[149] Zhou N., Zhao Y., Yao Y., Wu N., Xu M., Du H., Wu J., Tu Y. (2022). Antioxidant Stress and Anti-Inflammatory Activities of Egg White Proteins and Their Derived Peptides: A Review. J. Agric. Food Chem..

[150] Yao H., Zhao W., Zhao X., Fan R., Khoso P.A., Zhang Z., Liu W., Xu S. (2014). Selenium deficiency mainly influences the gene expressions of antioxidative selenoproteins in chicken muscles. Biol. Trace Elem. Res..

[151] Zhang J., Head B., Leonard S.W., Choi J., Tanguay R.L., Traber M.G. (2021). Vitamin E deficiency dysregulates thiols, amino acids and related molecules during zebrafish embryogenesis. Redox Biol..

[152] Traber M.G. (2021). Vitamin E: Necessary nutrient for neural development and cognitive function. Proc. Nutr. Soc..

[153] Zhang Q.-F., Li Y.-W., Liu Z.-H., Chen Q.-L. (2016). Reproductive toxicity of inorganic mercury exposure in adult zebrafish: Histological damage, oxidative stress, and alterations of sex hormone and gene expression in the hypothalamic-pituitary-gonadal axis. Aquat. Toxicol..

[154] Liu W.-C., Zhuang D.-P., Zhao Y., Balasubramanian B., Zhao Z.-H. (2022). Seaweed-Derived Polysaccharides Attenuate Heat Stress-Induced Splenic Oxidative Stress and Inflammatory Response via Regulating Nrf2 and NF-κB Signaling Pathways. Mar. Drugs.

[155] Chen H., Wang F., Wu X., Yuan S., Dong H., Zhou C., Feng S., Zhao Z., Si L. (2024). Chronic Heat Stress Induces Oxidative Stress and Induces Inflammatory Injury in Broiler Spleen via TLRs/MyD88/NF-κB Signaling Pathway in Broilers. Vet. Sci..

[156] Ye X.-Q., Zhu Y.-R., Yang Y.-Y., Qiu S.-J., Liu W.-C. (2023). Biogenic Selenium Nanoparticles Synthesized with Alginate Oligosaccharides Alleviate Heat Stress-Induced Oxidative Damage to Organs in Broilers through Activating Nrf2-Mediated Anti-Oxidation and Anti-Ferroptosis Pathways. Antioxidants.

[157] Zhao Z.-X., Yuan Y.-M., Zhao Z.-H., Yao Q.-H., Ye X.-Q., Wang Y.-Y., Liu H.-M., Jha R., Balasubramanian B., Liu W.-C. (2024). Phlorotannin Alleviates Liver Injury by Regulating Redox Balance, Apoptosis, and Ferroptosis of Broilers under Heat Stress. Antioxidants.

[158] Wang H., Jiang C., Xu B., Lei D., Fang R., Tang Y. (2025). Transcriptomic analysis revealed ferroptosis in ducklings with splenic necrosis induced by NDRV infection. Vet. Res..

[159] Zhu Y., Ma X.-Y., Cui L.-G., Xu Y.-R., Li C.-X., Talukder M., Li X.-N., Li J.-L. (2024). Di (2-ethylhexyl) phthalate induced lipophagy-related renal ferroptosis in quail (*Coturnix japonica*). Sci. Total Environ..

[160] Distéfano A.M., Martin M.V., Córdoba J.P., Bellido A.M., D’Ippólito S., Colman S.L., Soto D., Roldán J.A., Bartoli C.G., Zabaleta E.J. (2017). Heat stress induces ferroptosis-like cell death in plants. J. Cell Biol..

[161] Sánchez-Sanuy F., Mateluna-Cuadra R., Tomita K., Okada K., Sacchi G.A., Campo S., San Segundo B. (2022). Iron Induces Resistance Against the Rice Blast Fungus Magnaporthe oryzae Through Potentiation of Immune Responses. Rice.

[162] Xiong X., Zeng J., Ning Q., Liu H., Bu Z., Zhang X., Zeng J., Zhuo R., Cui K., Qin Z. (2024). Ferroptosis induction in host rice by endophyte OsiSh-2 is necessary for mutualism and disease resistance in symbiosis. Nat. Commun..

[163] Macharia M., Das P.P., Naqvi N.I., Wong S.-M. (2020). iTRAQ-based quantitative proteomics reveals a ferroptosis-like programmed cell death in plants infected by a highly virulent tobacco mosaic virus mutant 24A+UPD. Phytopathol. Res..

[164] Kalaipandian S., Powell J., Karunakaran A., Stiller J., Adkins S., Kage U., Kazan K., Fleury D. (2023). Transcriptome Analysis of Heat Shock Factor C2a Over-Expressing Wheat Roots Reveals Ferroptosis-like Cell Death in Heat Stress Recovery. Int. J. Mol. Sci..

[165] Chen X., Wu G., Dang Y.-X., Li Q., Xie M.-T., Li W., Zhang H., Lai J.-L. (2023). Uranium triggers ferroptosis-like cell death in Vicia faba roots by increasing iron accumulation and inhibiting glutathione peroxidase activity. Environ. Exp. Bot..

[166] Distéfano A.M., Bauer V., Cascallares M., López G.A., Fiol D.F., Zabaleta E., Pagnussat G.C. (2025). Heat stress in plants: Sensing, signalling, and ferroptosis. J. Exp. Bot..

[167] Shen Q., Naqvi N.I. (2024). The Ferroptosis landscape of biotic interactions in plants. Curr. Opin. Plant Biol..

[168] Jwa N.-S., Hwang B.K. (2025). Ferroptosis in plant immunity. Plant Commun..

[169] Aguilera A., Berdun F., Bartoli C., Steelheart C., Alegre M., Bayir H., Tyurina Y.Y., Kagan V.E., Salerno G., Pagnussat G. (2022). C-ferroptosis is an iron-dependent form of regulated cell death in cyanobacteria. J. Cell Biol..

[170] Srinivasan R., Han H.-S., Subramanian P., Mageswari A., Kim S.-H., Tirumani S., Maurya V.K., Muthukaliannan G.K., Ramya M. (2023). Lipid ROS- and Iron-Dependent Ferroptotic Cell Death in Unicellular Algae Chlamydomonas reinhardtii. Cells.

[171] Shen Q., Liang M., Yang F., Deng Y.Z., Naqvi N.I. (2020). Ferroptosis contributes to developmental cell death in rice blast. New Phytol..

[172] Conrad M., Kagan V.E., Bayir H., Pagnussat G.C., Head B., Traber M.G., Stockwell B.R. (2018). Regulation of lipid peroxidation and ferroptosis in diverse species. Genes Dev..

[173] Berndt C., Alborzinia H., Amen V.S., Ayton S., Barayeu U., Bartelt A., Bayir H., Bebber C.M., Birsoy K., Böttcher J.P. (2024). Ferroptosis in health and disease. Redox Biol..

[174] Dai E., Chen X., Linkermann A., Jiang X., Kang R., Kagan V.E., Bayir H., Yang W.S., Garcia-Saez A.J., Ioannou M.S. (2024). A guideline on the molecular ecosystem regulating ferroptosis. Nat. Cell Biol..

[175] Luan X., Chen P., Miao L., Yuan X., Yu C., Di G. (2025). Ferroptosis in organ ischemia-reperfusion injuries: Recent advancements and strategies. Mol. Cell Biochem..

[176] Eggenhofer E., Proneth B. (2025). Ferroptosis Inhibition: A Key Opportunity for the Treatment of Ischemia/Reperfusion Injury in Liver Transplantation. Transplantation.

[177] Hadian K., Stockwell B.R. (2023). The therapeutic potential of targeting regulated non-apoptotic cell death. Nat. Rev. Drug Discov..

[178] Du B., Fu Q., Yang Q., Yang Y., Li R., Yang X., Yang Q., Li S., Tian J., Liu H. (2025). Different types of cell death and their interactions in myocardial ischemia-reperfusion injury. Cell Death Discov..

[179] Shi Z., Du Y., Zheng J., Tang W., Liang Q., Zheng Z., Liu B., Sun H., Wang K., Shao C. (2024). Liproxstatin-1 Alleviated Ischemia/Reperfusion-Induced Acute Kidney Injury via Inhibiting Ferroptosis. Antioxidants.

[180] Lv Q., Lin J., Huang H., Ma B., Li W., Chen J., Wang M., Wang X., Fu G., Xiao Y. (2024). Nanosponge for Iron Chelation and Efflux: A Ferroptosis-Inhibiting Approach for Myocardial Infarction Therapy. Adv. Sci..

[181] Tonnus W., Maremonti F., Gavali S., Schlecht M.N., Gembardt F., Belavgeni A., Leinung N., Flade K., Bethe N., Traikov S. (2025). Multiple oestradiol functions inhibit ferroptosis and acute kidney injury. Nature.

[182] Wang R., Nie W., Yan X., Luo K., Zhang Q., Wang T., Lu E., Chen Y., Luo Y., Zhang Z. (2025). Biomimetic Nanomotors for Deep Ischemia Penetration and Ferroptosis Inhibition in Neuroprotective Therapy of Ischemic Stroke. Adv. Mater..

[183] Nguyen T.P.M., Alves F., Lane D.J.R., Bush A.I., Ayton S. (2025). Triggering ferroptosis in neurodegenerative diseases. Trends Neurosci..

[184] Wang Y., Li H., He Q., Zou R., Cai J., Zhang L. (2024). Ferroptosis: Underlying mechanisms and involvement in neurodegenerative diseases. Apoptosis.

[185] Su Z., Kon N., Yi J., Zhao H., Zhang W., Tang Q., Li H., Kobayashi H., Li Z., Duan S. (2023). Specific regulation of BACH1 by the hotspot mutant p53R175H reveals a distinct gain-of-function mechanism. Nat. Cancer.

[186] Zhang C., Liu X., Jin S., Chen Y., Guo R. (2022). Ferroptosis in cancer therapy: A novel approach to reversing drug resistance. Mol. Cancer.

[187] Liu T., Zhu C., Chen X., Guan G., Zou C., Shen S., Wu J., Wang Y., Lin Z., Chen L. (2022). Ferroptosis, as the most enriched programmed cell death process in glioma, induces immunosuppression and immunotherapy resistance. Neuro Oncol..

[188] Schwab A., Rao Z., Zhang J., Gollowitzer A., Siebenkäs K., Bindel N., D’Avanzo E., van Roey R., Hajjaj Y., Özel E. (2024). Zeb1 mediates EMT/plasticity-associated ferroptosis sensitivity in cancer cells by regulating lipogenic enzyme expression and phospholipid composition. Nat. Cell Biol..

[189] Guo W., Duan Z., Wu J., Zhou B.P. (2025). Epithelial-mesenchymal transition promotes metabolic reprogramming to suppress ferroptosis. Semin. Cancer Biol..

[190] Sendtner N., Seitz R., Brandl N., Müller M., Gülow K. (2025). Reactive Oxygen Species Across Death Pathways: Gatekeepers of Apoptosis, Ferroptosis, Pyroptosis, Paraptosis, and Beyond. Int. J. Mol. Sci..

[191] Wang B., Wang Y., Zhang J., Hu C., Jiang J., Li Y., Peng Z. (2023). ROS-induced lipid peroxidation modulates cell death outcome: Mechanisms behind apoptosis, autophagy, and ferroptosis. Arch. Toxicol..

[192] Ren Y., Wang R., Weng S., Xu H., Zhang Y., Chen S., Liu S., Ba Y., Zhou Z., Luo P. (2023). Multifaceted role of redox pattern in the tumor immune microenvironment regarding autophagy and apoptosis. Mol. Cancer.

[193] Schenk B., Fulda S. (2015). Reactive oxygen species regulate Smac mimetic/TNFα-induced necroptotic signaling and cell death. Oncogene.

[194] Tschopp J., Schroder K. (2010). NLRP3 inflammasome activation: The convergence of multiple signalling pathways on ROS production?. Nat. Rev. Immunol..

[195] Villalpando-Rodriguez G.E., Gibson S.B. (2021). Reactive Oxygen Species (ROS) Regulates Different Types of Cell Death by Acting as a Rheostat. Oxid. Med. Cell Longev..

[196] Hong S.H., Lee D.-H., Lee Y.-S., Jo M.J., Jeong Y.A., Kwon W.T., Choudry H.A., Bartlett D.L., Lee Y.J. (2017). Molecular crosstalk between ferroptosis and apoptosis: Emerging role of ER stress-induced p53-independent PUMA expression. Oncotarget.

[197] Andrysik Z., Espinosa J.M. (2025). Harnessing p53 for targeted cancer therapy: New advances and future directions. Transcription.

[198] Liu Y., Stockwell B.R., Jiang X., Gu W. (2025). p53-regulated non-apoptotic cell death pathways and their relevance in cancer and other diseases. Nat. Rev. Mol. Cell Biol..

[199] Li Y.-J., Fahrmann J.F., Aftabizadeh M., Zhao Q., Tripathi S.C., Zhang C., Yuan Y., Ann D., Hanash S., Yu H. (2022). Fatty acid oxidation protects cancer cells from apoptosis by increasing mitochondrial membrane lipids. Cell Rep..

[200] Hohorst L., Ros U., Garcia-Saez A.J. (2025). Mitochondrial dynamics and pore formation in regulated cell death pathways. Trends Biochem. Sci..

[201] Shen M., Cao S., Long X., Xiao L., Yang L., Zhang P., Li L., Chen F., Lei T., Gao H. (2024). DNAJC12 causes breast cancer chemotherapy resistance by repressing doxorubicin-induced ferroptosis and apoptosis via activation of AKT. Redox Biol..

[202] Tonnus W., Meyer C., Steinebach C., Belavgeni A., von Mässenhausen A., Gonzalez N.Z., Maremonti F., Gembardt F., Himmerkus N., Latk M. (2021). Dysfunction of the key ferroptosis-surveilling systems hypersensitizes mice to tubular necrosis during acute kidney injury. Nat. Commun..

[203] Zhao P., Yin S., Qiu Y., Sun C., Yu H. (2025). Ferroptosis and pyroptosis are connected through autophagy: A new perspective of overcoming drug resistance. Mol. Cancer.

[204] Wen Q., Liu J., Kang R., Zhou B., Tang D. (2019). The release and activity of HMGB1 in ferroptosis. Biochem. Biophys. Res. Commun..

[205] Chen R., Zou J., Liu J., Kang R., Tang D. (2025). DAMPs in the immunogenicity of cell death. Mol. Cell.

[206] Gao W., Huang Z., Duan J., Nice E.C., Lin J., Huang C. (2021). Elesclomol induces copper-dependent ferroptosis in colorectal cancer cells via degradation of ATP7A. Mol. Oncol..

[207] Malla S., Neupane R., Sood S., Hussein N., Abou-Dahech M., Terrero D., Ashby C.R., Babu R.J., Tiwari A.K. (2025). Mitochondria as Regulators of Nonapoptotic Cell Death in Cancer. MedComm (2020).

[208] von Krusenstiern A.N., Robson R.N., Qian N., Qiu B., Hu F., Reznik E., Smith N., Zandkarimi F., Estes V.M., Dupont M. (2023). Identification of essential sites of lipid peroxidation in ferroptosis. Nat. Chem. Biol..

[209] Hirata Y., Cai R., Volchuk A., Steinberg B.E., Saito Y., Matsuzawa A., Grinstein S., Freeman S.A. (2023). Lipid peroxidation increases membrane tension, Piezo1 gating, and cation permeability to execute ferroptosis. Curr. Biol..

[210] Pedrera L., Espiritu R.A., Ros U., Weber J., Schmitt A., Stroh J., Hailfinger S., von Karstedt S., García-Sáez A.J. (2021). Ferroptotic pores induce Ca2+ fluxes and ESCRT-III activation to modulate cell death kinetics. Cell Death Differ..

[211] Acoba M.G., Senoo N., Claypool S.M. (2020). Phospholipid ebb and flow makes mitochondria go. J. Cell Biol..

[212] Zhang D.D. (2024). Ironing out the details of ferroptosis. Nat. Cell Biol..

[213] Lee J., Roh J.-L. (2025). Ferroptosis: Iron release mechanisms in the bioenergetic process. Cancer Metastasis Rev..

[214] Zhang X., Tang B., Luo J., Yang Y., Weng Q., Fang S., Zhao Z., Tu J., Chen M., Ji J. (2024). Cuproptosis, ferroptosis and PANoptosis in tumor immune microenvironment remodeling and immunotherapy: Culprits or new hope. Mol. Cancer.

[215] Wang W., Green M., Choi J.E., Gijón M., Kennedy P.D., Johnson J.K., Liao P., Lang X., Kryczek I., Sell A. (2019). CD8+ T cells regulate tumour ferroptosis during cancer immunotherapy. Nature.

[216] Tang B., Zhu J., Wang Y., Chen W., Fang S., Mao W., Xu Z., Yang Y., Weng Q., Zhao Z. (2023). Targeted xCT-mediated Ferroptosis and Protumoral Polarization of Macrophages Is Effective against HCC and Enhances the Efficacy of the Anti-PD-1/L1 Response. Adv. Sci..

[217] Xu C., Sun S., Johnson T., Qi R., Zhang S., Zhang J., Yang K. (2021). The glutathione peroxidase Gpx4 prevents lipid peroxidation and ferroptosis to sustain Treg cell activation and suppression of antitumor immunity. Cell Rep..

[218] Wu J., Feng Z., Chen L., Li Y., Bian H., Geng J., Zheng Z.-H., Fu X., Pei Z., Qin Y. (2022). TNF antagonist sensitizes synovial fibroblasts to ferroptotic cell death in collagen-induced arthritis mouse models. Nat. Commun..

